# Phytochemical analysis, antioxidant, anti-inflammatory and enzyme inhibitory activities of bean pear (*Pyrus calleryana* fruit)

**DOI:** 10.3389/fpls.2025.1521990

**Published:** 2025-02-07

**Authors:** Huan Zhao, Qinqin Wang, Lanlan Yang, Yuanquan Ran, Qiong Hu, Yi Hong, Minyi Tian

**Affiliations:** ^1^ Key Laboratory of Plant Resource Conservation and Germplasm Innovation in Mountainous Region (Ministry of Education), School of Liquor and Food Engineering, Guizhou University, Guiyang, China; ^2^ National & Local Joint Engineering Research Center for the Exploitation of Homology Resources of Southwest Medicine and Food, Guizhou University, Guiyang, China; ^3^ First Affiliated Hospital of Guizhou University of Traditional Chinese Medicine, Guiyang, China

**Keywords:** *Pyrus calleryana*, chemical components, antioxidant, anti-inflammatory effect, enzyme inhibitory activity

## Abstract

*Pyrus calleryana* fruit (bean pear) is processed into fruit wine and used in traditional Chinese medicine. The present study reported phytochemical constituents, antioxidant, anti-inflammatory, and enzyme inhibitory activities of *P. calleryana* fruit water extract (WE) and ethanol extract (EE). In the *P. calleryana* fruit WE and EE, 63 compounds were identified using UHPLC-Q-Orbitrap-MS analysis, including 23 phenols, 13 flavonoids, 14 terpenoids, and 13 other types of compounds. In the antioxidant activity, WE and EE showed marked free radical scavenging effects on both ABTS (2.33 ± 0.15 μg/mL and 2.23 ± 0.15 μg/mL, respectively) and DPPH (5.93 ± 0.55 μg/mL and 7.07 ± 0.23 μg/mL, respectively), especially, their scavenging effects on DPPH free radicals were superior or equivalent to that of BHT (7.47 ± 0.47 μg/mL). In LPS-induced RAW264.7 cells, *P. calleryana* fruit WE and EE remarkably inhibited the secretion of inflammatory factors, and the inhibitory effect of WE on the release of IL-6, NO, and PGE_2_ was superior or equivalent to that of EE. Interestingly, *P. calleryana* fruit WE and EE exhibited potent inhibition on α-glucosidase (0.60 ± 0.09 μg/mL and 0.48 ± 0.09 μg/mL, respectively) and tyrosinase (210.11 ± 2.59 μg/mL and 45.35 ± 0.96 μg/mL, respectively), which were superior to their respective positive controls acarbose (302.57 ± 22.09 μg/mL) and arbutin (243.07 ± 15.91 μg/mL). Our findings suggested that *P. calleryana* fruit WE and EE possess significant antioxidant, anti-inflammatory, α-glucosidase, and tyrosinase inhibitory properties. Thus, *P. calleryana* fruit has great potential for application in functional food products.

## 1 Introduction

*Pyrus calleryana* Decne., a deciduous tree of the genus *Pyrus* in the Rosaceae family, is a native tree species of China (Sapkota et al., 2022) and primarily distributed in Zhejiang, Jiangsu, Jiangxi, Hubei, Guangdong, Guangxi, Yunnan, and Guizhou provinces in China (Jiang et al., 2016). *P. calleryana* fruit, commonly known as bean pear, is rich in polysaccharides (15-20%) (Liu et al., 2011). It is often eaten after cooking, primarily due to its sour and astringent when raw, and cooking not only eliminates its sour and astringent taste but also makes it very sweet. Besides, it is also a common raw material for brewing fruit wine (Wu and Wu, 2012).

In addition, *P. calleryana* is often used as a rootstock for pear (*Pyrus pyrifolia*), and its wood is employed to make furniture (Li et al., 2015). It is also cultivated as an ornamental tree due to its bright white flowers in early spring (Culley and Hardiman, 2007; Santamour and Demuth, 1980; Sapkota et al., 2022). As a folk medicine, various parts of *P. calleryana*, including its fruit, branches, leaf, and root, are used medicinally to treat stomach pain, cough, indigestion, acute conjunctivitis, and dysentery (China Medical Information Platform, 2024). Past studies of phytochemical constituents and pharmacological activity of *P. calleryana* have shown that its leaf ethanol extract exhibited strong antioxidant effects (Nassar et al., 2011), and the volatile oil of its fruit possessed antibacterial and anti-inflammatory effects (Tian et al., 2023).

*P. calleryana* fruit possesses both edible and medicinal values and has great development potential in functional foods. Nevertheless, there are fewer studies on its chemical composition and bioactivities. Hence, in this study, we aimed to analyze the phytochemical composition of *P. calleryana* fruit WE and EE, explore their antioxidant, anti-inflammatory effects, and enzyme inhibitory activities, and offer a theoretical basis for exploiting *P. calleryana* fruit in the field of functional foods.

## 2 Materials and methods

### 2.1 Chemical and reagents

RAW264.7 cells were purchased at the Kunming Cell Bank (Kunming, China). Folin-Ciocalteu reagent, rutin, arbutin, lipopolysaccharide (LPS), 3-[4,5-Dimethylthiazol-2-yl]-2, 5-diphenyltetrazolium bromide (MTT), dexamethasone (DXM), galanthamine, dimethyl sulfoxide (DMSO), acarbose and gallic acid were supplied from Solarbio Sciences & Technology (Beijing, China). Mouse TNF-α and IL-6 ELISA kits were obtained from Multi Sciences (Lianke) Biotech, Co., Ltd. (Hangzhou, China). Abnova Corporation (Taiwan, China) provided a prostaglandin E_2_ (PGE_2_) ELISA kit. Ascorbic acid, 5,5’dithiobis-(2-nitrobenzoic acid), tyrosinase, p-nitrophenyl-α-D-glucopyranoside (p-NPG), L-tyrosine, acetylthiocholine iodide (ATCI), acetylcholinesterase (AChE), butyrylthiocholine chloride (BTCl), butyrylcholinesterase (BChE), butylated hydroxytoluene (BHT), 2,2-azino-bis-3-ethylbenzthiazoline-6-sulphonic acid (ABTS), α-glucosidase, and 1,1-Diphenyl-2-picrylhydrazyl (DPPH) were supplied by Sigma-Aldrich (Germany). Beyotime Biotechnology offered the NO detection kit (Shanghai, China).

### 2.2 Plant material

*P. calleryana* fruit was bought from Shansen Reservoir, Yudu County, Ganzhou City, Jiangxi Province, and was identified by Prof. Guoxiong Hu, and preserved in the College of Life Sciences, Guizhou University (herbarium number: PC20210907).

### 2.3 Extraction of *P. calleryana* fruit WE and EE

Fresh *P. calleryana* fruit was chopped, placed in a round-bottomed flask, and extracted with ultrapure water or 70% ethanol solution at a material-liquid ratio of 1:3 (w/v) for 2 h. Afterward, the filtrate was gathered, and the residue was re-extracted according to the above conditions. Subsequently, the two filtrates were merged and condensed by rotary evaporation to obtain the concentrated solution. Finally, the concentrated solution was dried by a freeze-dryer to obtain the WE and EE. The samples were packed into brown bottles and sealed storage at 4°C.

### 2.4 Chemical composition analysis

Ultra-high-performance liquid chromatography coupled to quadrupole-Orbitrap high-resolution mass spectrometry (UHPLC-Q-Orbitrap-MS) was used to identify phytochemical components of *P. calleryana* fruit WE and EE. Dionex Ultimate 3000 RSLC liquid phase conditions were as follows: injection volume (5 μL), column (Thermo Fisher Hypersil GOLD aQ, 2.1 mm × 100 mm, 1.9 μm), column temperature (40°C), mobile phase (A: 0.1% formic acid aqueous solution and B: 0.1% formic acid acetonitrile), and flow rate (0.3 mL/min). The gradient elution conditions were as follows: 0~2 min (5% B), 2~42 min (5~95% B), 42~47 min (95% B), 47~47.1 min (5% B), 47.1~50 min (5% B).

Thermo Fisher Scientific’s Q Exactive Focus hybrid quadrupole-Orbitrap high-resolution mass spectrometry (Q-Orbitrap-MS) with heated electrospray ionization (HESI-II) was used to acquire MS data. The HESI-II settings were as follows: sheath gas (35 arb), auxiliary gas (10 arb), S-Lens (60), probe heater temperature (350°C), spray voltages (-2.5/+3.0 kV), capillary temperature (320°C), sweep gas (0 arb). The mass spectrometry scanning parameters were as follows: full MS scan range (100 to 1500 m/z), loop count (3), intensity threshold (1.6 e^5^), stepped NCE (20, 40, 60 eV), maximum injection time (full MS: 100 ms, MS/MS: 50 ms), resolution (full MS: 70,000, MS/MS: 17,500), AGC target (full MS: 1 e^6^, MS/MS: 2 e^5^). Thermo Fisher Scientific Xcalibur 4.1 (Waltham, MA, USA) was utilized for the processing of mass spectrometry data on chemical compositions. Phytochemicals were identified by comparing the mzVault and mzCloud databases and references within a 10 ppm threshold.

### 2.5 Determination of total phenolic acid content and total flavonoid content

#### 2.5.1 Determination of TPC

The Folin-Ciocalteu method was utilized to determine the TPC in the *P. calleryana* fruit WE and EE, with gallic acid as the standard. WE or EE were dissolved in ultrapure water to prepare sample solution. Folin-Ciocalteu solution (2.5 mL) and sample solution (0.5 mL) were accurately pipetted into a 5 mL tube and reacted for 4 min. Afterward, 7.5% Na_2_CO_3_ solution (2 mL) was added, mixed, and reacted for 60 min at room temperature. After recording the absorbance at 760 nm, we used linear fitting to create a standard curve with the absorbance values as the vertical coordinate and the concentration of gallic acid as the horizontal coordinate. The TPC of *P. calleryana* fruit WE and EE was calculated according to the standard curve and expressed as milligrams of gallic acid equivalents per gram of sample (mg GAEs/g sample).

#### 2.5.2 Determination of TFC

The TFC in the WE and EE of *P. calleryana* fruit was estimated by the NaNO_2_-Al(NO_3_)_3_-NaOH colorimetric method and rutin was used as a standard. The WE or EE dissolved in 70% ethanol (5 mL) and NaNO_2_ solution (5%, 0.4 mL) were mixed and reacted for 6 min. Next, we added Al(NO_3_)_3_ solution (10%, 0.4 mL) and let it react for 6 min. Subsequently, NaOH solution (4%, 4 mL) and ultrapure water (0.2 mL) were added and reacted for 15 min. Then, the absorbance at 510 nm was measured. The standard curve was established using rutin concentration as the horizontal coordinate and absorbance as the vertical coordinate. The standard curve was used to calculate the TFC, which was then expressed as milligrams of rutin equivalents per gram of sample (mg REs/g sample).

### 2.6 Measurement of antioxidant capacity of *P. calleryana* fruit WE and EE

The ability of *P. calleryana* fruit WE and EE to scavenge free radicals was determined by ABTS and DPPH assay. Ascorbic acid and BHT were used as positive controls.

#### 2.6.1 ABTS assay

In the ABTS free radical scavenging capacity assay, ABTS solution (0.7 mM) was prepared by dissolving ABTS (19.2 mg) with dehydrated ethanol (50 mL), and K_2_S_2_O_4_ solution (2.4 mM) was obtained by dissolving K_2_S_2_O_4_ (33 mg) with ultrapure water (50 mL). Subsequently, equal volumes of ABTS and K_2_S_2_O_4_ solution were mixed, protected from light, and incubated at 37°C for 12 h to prepare ABTS•^+^ solution. The ABTS assay included a sample group, a control group, and a blank group. Sample solution (18 μL) was combined with ABTS•^+^ solution (180 μL) in the sample group; in the control group, sample solution (18 μL) was combined with solvent of sample solution (180 μL); in the blank group, 18 μL of the solvent of sample solution and 180 μL of ABTS•^+^ solution were added. Then, all groups were incubated for 10 min and sheltered from light. The absorbance (A) was detected at 734 nm. The findings were displayed as milligrams of ascorbic acid equivalents per gram of sample (mg AEs/g sample) and IC_50_ values. The following formula was used to determine the scavenging rate:


$$\text{Scavenging rate}(\%)=[1-\frac{{\text{A}}_{\text{sample}}-{\text{A}}_{\text{control}}\;}{\;{\text{A}}_{\text{blank}}}]\times 100\%$$

#### 2.6.2 DPPH assay

In the free radical clearance test for DPPH, DPPH (0.08 mM) solution was prepared by dissolving DPPH (3.15 mg) in dehydrated ethanol (100 mL). The sample group, control group, and blank group were set up. In the sample group, the sample solution (100 μL) was mixed with an equal amount of DPPH solution. Similarly, equal volumes of sample solution and solvent of sample solution were mixed as the control group. In the blank group, the DPPH solution and solvent of the sample solution were mixed in a 1:1 ratio (v/v). Next, these groups were placed under room temperature and light-avoidance conditions for 0.5 h, and their absorbance values were detected at 517 nm. The free radical scavenging capacity was expressed as milligrams of ascorbic acid equivalents per gram of sample (mg AEs/g sample) and IC_50_ values. The aforementioned formula was used to determine the scavenging rate.

### 2.7 Anti-inflammatory capacity analysis in LPS-induced RAW264.7 cells

#### 2.7.1 Analysis of cytotoxicity

MTT test was utilized to test the toxicity of *P. calleryana* fruit WE and EE on RAW264.7 cells (Zhao et al., 2020a). The samples were dissolved in DMSO and diluted half-fold with culture medium (DMSO content was less than 0.05%). Cells (2×10^5^ cells/well) were inoculated and cultured in 96-well plates. Then, the old culture medium was aspirated out and discarded after 24 h incubation. Afterward, the sample solution was pipetted into 96 well plates and incubated for 24 h at 37°C. Next, MTT solution (10 μL, 5 mg/mL) was pipetted into and reacted for 4 h. Subsequently, the old supernatant was discarded, and 150 μL of DMSO was added to each well. Finally, the 96-well plate was shaken at room temperature for 10 min, and the absorbance value was determined at 490 nm.

#### 2.7.2 Measurement of NO, IL-6, TNF-α, and PGE_2_ release

In the assay of inflammatory factors release, 100 μL of cell suspensions (2×10^6^ cells/mL) were inoculated in 96-well plates and cultured for 24 h. The sample group, blank group, positive group (DXM), and model group (LPS) were set up. Then, the old supernatants were aspirated out and discarded. In the sample group, 100 μL of sample solution (62.5, 125, and 250 μg/mL) was added; 100 μL of DXM (20 μg/mL) was added in the positive group; 100 μL of culture medium was added in blank and model groups. After 2 h of incubation, the supernatants were discarded; in the sample group, equal volumes of sample solution (100 μL) and LPS (100 μL) were mixed, and the sample solution’s ultimate concentrations were 62.5, 125, and 250 μg/mL; DXM (100 μL) and LPS (100 μL) were added to the positive group, and the ultimate concentration of DXM was 20 μg/mL. In the model group, culture medium (100 μL) and LPS (100 μL) were mixed, and the three groups mentioned above had a final concentration of 1 μg/mL of LPS. In addition, culture medium (200 μL) was added to the blank group. Next, all the groups were placed at a constant temperature of 37°C for 24 h. Then, all the groups were photographed under an inverted fluorescence microscope to observe cell morphological changes. Subsequently, the cell supernatants were collected. Determination of NO release was performed referring to the instructions of the NO detection kit, and the levels of IL-6, TNF-α, and PGE_2_ were measured by ELISA kits.

### 2.8 Enzyme inhibitory activity assay

The inhibitory activities of *P. calleryana* fruit WE and EE on tyrosinase, α-glucosidase, and cholinesterase were determined by using arbutin, acarbose, and galanthamine as positive controls, respectively.

#### 2.8.1 Tyrosinase inhibitory activity

In the assay of tyrosinase inhibitory activity, we set up four groups: sample group, sample blank group, negative group, and blank group. First, sample solution (70 μL) was added to the sample and sample blank groups, and PBS buffer (70 μL, pH=6.8) was added in the negative and blank groups. Subsequently, tyrosinase (100 μL, 100 U/mL) was added in the sample and negative groups, while PBS buffer (100 μL, pH=6.8) was spiked into the sample blank and blank groups. The above four groups were incubated at a constant temperature of 37°C for 5 min. Then, L-tyrosine (80 μL, 5.5 mM) was added in all groups. After 37°C incubation for 30 min, their absorbance (A) was determined at 492 nm. The following formula was used to get the tyrosinase inhibition rate, and IC_50_ values were utilized to express the inhibitory activity of tyrosinase.


$$\text{Inhibition rate}(\%)=[1-\frac{{\text{A}}_{\text{sample}}-{\text{A}}_{\text{sample blank}}\;}{{\text{A}}_{\text{negative}}\;-{\text{A}}_{\text{blank}}}]\times 100\%$$

#### 2.8.2 α-Glucosidase inhibitory activity assay

In the α-glucosidase inhibitory activity assay, the experimental procedure was divided into four groups: sample group, sample blank group, negative group, and blank group. First, 90 μL of sample solution was added into the sample and sample blank group, whereas 90 μL of PBS buffer (pH=6.8) was utilized in the negative and blank groups. Next, 10 μL of α-glucosidase (0.8 U/mL) was added to the sample and negative groups, while 10 μL of PBS buffer (pH=6.8) was added to the sample blank and blank groups. The four groups were placed in a constant temperature incubator of 37°C for 15 min. Then, 10 μL of the p-NPG (1 mM) was added to all groups and reacted at 37°C for 15 min. Finally, the reaction was terminated by adding 80 μL of sodium carbonate solution (0.2 mM). The absorbance values of each group were measured at 405 nm. The α-glucosidase inhibition rate was calculated as above, and IC_50_ values represented the inhibitory activity of α-glucosidase.

#### 2.8.3 Cholinesterase inhibitory activity assay

In the determination of cholinesterase inhibitory activity, we also set up sample, sample blank, negative, and blank groups. First, the sample and sample blank groups were given a 50 μL sample solution, while 50 μL of PBS buffer (pH=8) was added to the negative and blank groups. Subsequently, 10 μL of AChE or BChE (0.5 U/mL) was pipetted into the sample and negative groups, and 10 μL of PBS buffer (pH=8) was added to the sample blank and blank groups, which were kept at 4°C for 5 min. Then, 20 μL of ATCI (2 mM) or BTCI (2 mM) and 5,5’dithiobis-(2-nitrobenzoic acid) solution (2 mM) was added to all groups and maintained at 37°C for 30 min. Absorbance values were determined at 405 nm, and the inhibition rate was calculated as above. Finally, the inhibitory activity of the WE and EE on cholinesterase were expressed as IC_50_ values.

### 2.9 Statistical analysis

In this study, three independent replications of each experiment were carried out, and their results were represented using mean ± standard deviation (SD). The significant difference was analyzed by one-way analysis of variance (ANOVA) with Fischer’s LSD *post-hoc* test or two-tailed unpaired t-test using IBM SPSS Statistics 25 software (*p* < 0.05 indicates a significant difference).

## 3 Results and discussion

### 3.1 Phytochemical components of *P. calleryana* fruit WE and EE

**Figure 1:**
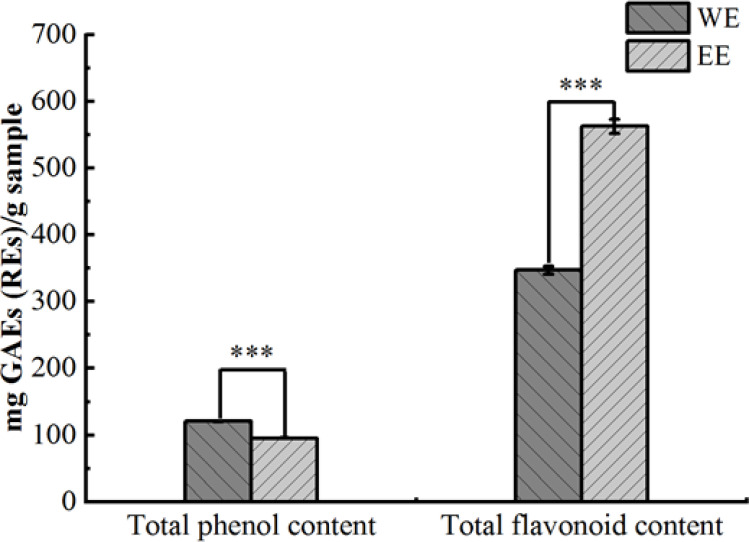
Total phenolic and flavonoid contents of *P. calleryana* fruit WE and EE, *** *p* < 0.001.

Based on fresh weight, the extraction rates of *P. calleryana* fruit WE and EE were 8.3% (w/w) and 11% (w/w), respectively. The Folin-Ciocalteu method and the NaNO_2_-Al(NO_3_)_3_-NaOH colorimetric method were used to determine the TPC and TFC of WE and EE, and the results are shown in **Figure 1**. The TPC of WE and EE were 120.74 ± 0.32 mg GAEs/g sample and 95.37 ± 0.28 mg GAEs/g sample, respectively, and WE exhibited more TPC compared to EE (*p* < 0.001). The TFC of WE and EE separately were 347.05 ± 6.27 mg REs/g sample and 562.51 ± 10.71 mg REs/g sample, and the TFC in EE was significantly higher than WE (*p* < 0.001).

Based on UHPLC-Q-Orbitrap-MS analysis, 63 compounds were characterized in the *P. calleryana* fruit WE and EE (detailed analysis of fragmentation patterns in **Supplementary Material**), of which 40 compounds were identified in WE, and 41 compounds were identified in EE (**Table 1**, **Supplementary Figure S1** in **Supplementary Materials**). A total of 23 phenolic compounds were identified as neochlorogenic acid (6), 3,5-dimethoxy-4-hydroxybenzaldehyde (8), gentisic acid (11), cryptochlorogenic acid (12), aloenin (14), chlorogenic acid (15), vanillic acid (16), 1-caffeoylquinic acid (17), caffeic acid (18), 7-hydroxycoumarin (21), vanillin (23), 2-hydroxy-4-methoxybenzaldehyde (24), protocatechualdehyde (28), ellagic acid (34), sinapyl aldehyde (36), dehydrodiisoeugenol (38), 3,5-dicaffeoylquinic acid (39), isochlorogenic acid B (40), isochlorogenic acid C (44, ethyl caffeate (49), astringin (51), 6-gingerol (53), and (+)-usniacin (60) (**Supplementary Figure S2**, **Supplementary Material**). Thirteen flavonoid compounds were identified, including (+)-catechin hydrate (22), procyanidin B1 (26), taxifolin 7-rhamnoside (29), medicarpin (31), hesperetin (32), morin (33), cynaroside (35), isorhamnetin (41), diosmetin-7-O-β-D-glucopyranoside (43), hyperoside (45), isoquercitrin (46), kaempferol-7-O-β-D-glucopyranoside (47), and luteolin (50) (**Supplementary Figure S3**, **Supplementary Material**). Fourteen terpenoid compounds were identified as ailanthone (20), sarracenin (25), perillene (37), germacrone (48), glabrolide (52), medicagenic acid (54), quillaic acid (55), 18β-glycyrrhetintic acid (56), ursonic acid (57), echinocystic acid (58), ursolic acid (59), acetyl-11-keto-β-boswellic acid (61), lupenone (62), and roburic acid (63) (**Supplementary Figure S4**, **Supplementary Material**). Besides, 13 other types of compounds were identified, including sucrose (1), quinic acid (2), γ-aminobutyric acid (3), L-tryptophan (4), scopolin (5), citric acid (7), o-veratraldehyde (9), mannitol (10), 2-isopropylmalic acid (13), homoveratrumic acid (19), ethyl 4-methoxycinnamate (27), androsin (30), and benzoic acid (42) (**Supplementary Figure S5**, **Supplementary Material**). Except for chlorogenic acid (15), vanillic acid (16), and caffeic acid (18), which were previously identified from *P. calleryana* leaf (Nassar et al., 2011), the remaining 60 compounds were identified for the first time in *P. calleryana*. The results showed that there were plenty of phenolic, flavonoid, and terpenoid compounds in *P. calleryana* fruit WE and EE.

**Table 1:** Chemical composition of *P. calleryana* fruit WE and EE were authenticated using UHPLC-Q-Orbitrap-MS in positive and negative ion modes.

Peak NO.	RT [min] ^a^	Identification ^b^	Formula	[M+H]^+^ (m/z)	[M-H]^-^ (m/z)	Error ppm ^c^	MS^2^ fragmentions	EE ^d^	WE ^d^
1	0.697	Sucrose	C_12_H_22_O_11_		341.10889	-0.1	119.03339, 101.02276, 89.02277, 71.01225, 59.01231		√
2	0.760	Quinic acid	C_7_H_12_O_6_		191.05548	-3.3	173.04387, 155.03320, 127.03832, 111.04366, 109.02768	√	√
3	0.910	γ-Aminobutyric acid	C_4_H_9_NO_2_	104.07079		1.8	87.04445, 86.09684, 60.08149, 58.06580		√
4	1.601	L-Tryptophan	C_11_H_12_N_2_O_2_	205.09665		-2.5	188.06987, 170.05904, 146.05960, 132.08040, 118.06502	√	√
5	1.804	Scopolin	C_16_H_18_O_9_	355.09998		-4.4	324.07999, 193.04649, 192.03851, 177.01558, 103.41244		√
6	1.832	Neochlorogenic acid	C_16_H_18_O_9_	355.09956		-4.3	324.08014, 193.04646, 192.03841, 163.03838, 135.04347	√	
7	1.897	Citric acid	C_6_H_8_O_7_		191.01820	-4.0	173.04372, 129.01775, 111.00710, 87.00710, 85.02784	√	√
8	1.916	3,5-Dimethoxy-4-hydroxybenzaldehyde	C_9_H_10_O_4_	183.06471		-2.6	140.04636, 135.04366, 123.04384, 121.02821, 95.04927	√	√
9	2.181	o-Veratraldehyde	C_9_H_10_O_3_	167.06992		-2.1	152.04637, 124.05170, 123.04391, 107.04926, 95.04931		√
10	2.601	Mannitol	C_6_H_14_O_6_		181.07097	-4.4	163.05931, 101.02270, 89.02272, 71.01222, 59.01227	√	
11	2.837	Gentisic acid	C_7_H_6_O_4_		153.01923	-0.7	125.02262, 109.02787, 108.02004, 91.01723		√
12	2.993	Cryptochlorogenic acid	C_16_H_18_O_9_		353.08624	-4.4	191.05457, 179.03334, 137.02298, 135.04344, 93.03290	√	√
13	3.957	2-Isopropylmalic acid	C_7_H_12_O_5_		175.06109	-0.6	157.04906, 115.03844, 113.05916, 85.06426		√
14	4.120	Aloenin	C_19_H_22_O_10_	411.12555		-4.7	381.11407, 249.07240, 203.05244, 185.04158, 126.15467	√	√
15	5.816	Chlorogenic acid	C_16_H_18_O_9_	355.10129		-3.0	235.05920, 163.03842, 145.02791, 135.04364, 117.03333		√
16	5.872	Vanillic acid	C_8_H_8_O_4_	169.04918		-2.1	125.05945, 123.04375, 111.04409, 110.03622, 93.03374		√
17	6.275	1-Caffeoylquinic acid	C_16_H_18_O_9_		353.08627	-4.3	191.05457, 173.04362, 161.02272, 135.04347, 127.03827	√	
18	6.411	Caffeic acid	C_9_H_8_O_4_		179.03422	-4.2	161.04358, 135.04352, 117.03301, 107.04850, 91.05380	√	√
19	7.311	Homoveratrumic acid	C_10_H_12_O_4_	197.08041		-2.2	179.06967, 151.03871, 125.05949, 95.04932, 77.03919		√
20	7.366	Ailanthone	C_20_H_24_O_7_	377.16147		4.1	359.07346, 215.05185, 197.04170, 163.03845, 117.03349		√
21	7.749	7-Hydroxycoumarin	C_9_H_6_O_3_	163.03856		-2.5	145.02802, 135.04373, 117.03342, 107.04918, 89.03891		√
22	8.257	(+)-Catechin hydrate	C_15_H_16_O_7_	289.07062	289.07062 [M-H_2_O-H]^-^	-3.9	245.08069, 203.06999, 151.03847, 125.02275, 109.02783	√	√
23	8.290	Vanillin	C_8_H_8_O_3_	153.05423		-2.6	135.04370, 125.05946, 111.04411,93.03373, 65.03922	√	
24	8.726	2-Hydroxy-4-methoxybenzaldehyde	C_8_H_8_O_3_	153.05420		-2.7	135.05406, 125.05947, 111.04412, 93.03374, 65.03921		√
25	8.829	Sarracenin	C_11_H_14_O_5_	227.09071		-3.0	209.08020, 181.04895, 155.06975, 140.04634, 123.04382		√
26	8.889	Procyanidin B1	C_30_H_26_O_12_		577.13287	-3.9	425.08621, 407.07541, 289.07065, 287.05432, 125.02273	√	√
27	9.094	Ethyl 4-methoxycinnamate	C_12_H_14_O_3_	207.10107		-2.4	163.11140, 151.03868, 133.02805, 107.04918, 95.04935		√
28	9.329	Protocatechualdehyde	C_7_H_6_O_3_	139.03859		-2.7	121.03928, 111.04404, 93.03373, 83.04951, 65.03915	√	
29	9.427	Taxifolin 7-rhamnoside	C_21_H_22_O_11_		449.10703	-4.2	287.05481, 269.04440, 259.06006, 151.00211, 125.02276	√	√
30	9.782	Androsin	C_15_H_2_0O_8_		327.10724	-4.0	165.05418, 147.04366, 123.00702, 89.02298, 71.01215	√	√
31	10.652	Medicarpin	C_16_H_14_O_4_	271.09570		-2.9	221.05888, 211.07466, 183.07986, 165.06943, 119.04896		√
32	11.981	Hesperetin	C_16_H_14_O_6_	303.08605		-0.9	257.04321, 247.05901, 229.04872, 153.01765, 137.02293	√	
33	12.108	Morin	C_15_H_10_O_7_	303.04892		-3.3	257.04321, 229.04872, 165.01768, 153.01765, 137.02293	√	
34	12.179	Ellagic acid	C_14_ H_6_ O_8_		300.99768	-4.4	283.99512, 229.01283, 185.02283, 173.02275, 145.02789		√
35	12.395	Cynaroside	C_21_H_20_O_11_	449.10675		-2.4	287.05405, 269.04492, 241.04945, 153.01765, 135.04372	√	√
36	12.711	Sinapyl aldehyde	C_11_H_12_O_4_	209.08017		-3.2	177.05339, 149.05872, 121.06417, 103.05393, 91.05415	√	
37	12.765	Perillene	C_10_H_14_O	151.11140		-2.3	123.04391, 105.03361, 95.04937, 79.05468, 67.05482		√
38	12.871	Dehydrodiisoeugenol	C_20_H_22_O_4_	327.15817		-2.8	263.10498, 163.07475, 151.07483, 137.05925, 122.03613	√	√
39	13.152	3,5-Dicaffeoylquinic acid	C_25_H_24_O_12_		515.11719	-4.3	353.08624, 191.05461, 179.03339, 161.02277, 135.04347	√	
40	13.172	Isochlorogenic acid B	C_25_H_24_O_12_		515.11731	-4.3	353.08621, 191.05458, 179.03339, 173.04384, 135.04347		√
41	13.430	Isorhamnetin	C_16_H_12_O_7_	317.06532		-0.8	302.04095, 285.03799, 257.04312, 165.01784, 153.01759	√	
42	13.595	Benzoic acid	C_7_H_6_O_2_	123.04391		-1.2	105.03352, 95.04929, 77.03898	√	√
43	13.797	Diosmetin-7-O-β-D-glucopyranoside	C_22_H_22_O_11_	463.12198		-3.3	301.06955, 286.04602, 258.05124, 229.04784, 137.05865	√	
44	13.816	Isochlorogenic acid C	C_25_H_24_O_12_		515.11761	-3.7	353.08612, 191.05453, 179.03336, 155.03325, 111.04331	√	√
45	14.084	Hyperoside	C_21_H_20_O_12_		463.08792	-0.6	301.07047, 255.02884, 179.03346, 151.00211, 135.04352		√
46	14.090	Isoquercitrin	C_21_H_20_O_12_		463.08908	1.9	301.07062, 271.02444, 179.03343, 161.02264, 135.04349	√	√
47	14.234	Kaempferol-7-O-β-D-glucopyranoside	C_21_H_20_O_11_		447.09421	2.1	285.07654, 179.03343, 135.04350, 133.02780, 107.04848		√
48	15.194	Germacrone	C_15_H_22_O	219.17363		-3.2	189.09038, 133.10081, 105.06994, 95.08569, 81.07027		√
49	15.913	Ethyl caffeate	C_11_H_12_O_4_		207.06582	-2.2	179.03339, 161.02281, 135.04350, 133.02782, 93.03288	√	
50	16.003	Luteolin	C_15_H_10_O_6_		285.03922	-4.4	199.03842, 175.03860, 151.00198, 133.02783, 107.01216	√	√
51	16.365	Astringin	C_20_H_22_O_9_		405.11728	-4.5	243.06490, 152.00984, 123.00716, 121.02779, 108.01981		√
52	20.632	Glabrolide	C_30_H_44_O_4_	469.32993		-2.8	451.31705, 433.30838, 405.31375, 231.16273, 119.08539	√	
53	22.089	6-Gingerol	C_17_H_26_O_4_		293.17459	-4.2	236.10402, 221.15329, 220.14540, 205.12187, 177.09055	√	√
54	22.159	Medicagenic acid	C_30_H_46_O_6_		501.31998	-4.4	483.30927, 471.30936, 453.29984, 439.32141, 409.31134	√	
55	26.831	Quillaic acid	C_30_H_46_O_5_		485.32520	-4.2	467.31467, 455.31393, 425.30414, 393.31271, 391.29834	√	
56	28.373	18β-Glycyrrhetintic acid	C_30_H_46_O_4_	471.34579		-2.3	425.34113, 271.20554, 235.16798, 189.16296, 175.14737	√	
57	28.818	Ursonic acid	C_30_H_46_O_3_	437.34021 [M-H_2_O+H]^+^		-2.7	437.34045, 409.34479, 391.33392, 205.15816, 203.17882,	√	
58	28.836	Echinocystic acid	C_30_H_48_O_4_		471.34583	-4.6	453.33603, 423.32571, 397.30945, 393.31378	√	
59	29.387	Ursolic acid	C_30_H_48_O_3_	457.36652		-2.4	439.35718, 249.18463, 235.16855, 189.16331, 119.08543	√	
60	30.645	(+)-Usniacin	C_18_H_16_O_7_		343.08081	-4.4	328.05753, 313.03430, 259.05981, 231.06496, 215.03368	√	
61	32.914	Acetyl-11-keto-β-boswellic acid	C_32_H_48_O_5_	513.35632		-2.2	407.32877, 271.20462, 235.16905, 217.15800, 189.16312	√	
62	36.211	Lupenone	C_30_H_48_O	407.36612 [M-H_2_O+H]^+^		-2.7	407.36685, 217.19466, 215.17896, 191.17868, 163.14748	√	
63	40.020	Roburic acid	C_30_H_48_O_2_	441.37149		-2.8	287.23599, 235.16869, 189.16298, 149.09579, 121.10110	√	

^a^ RT, Retention time. ^b^ Identification: Phytochemical compounds were identified by comparing the MS1 and MS2 fragments with the data in the mzVault and mzCloud databases and references. In addition, the detailed analysis of fragmentation patterns is presented in the **Supplementary Material**. ^c^ Error ppm represents mass error in parts per million (ppm) and was obtained from Thermo Fisher Scientific Xcalibur 4.1 software. ^d^ “√” means detected from extracts, “-” means undetected from extracts.

### 3.2 Antioxidant effects of *P. calleryana* fruit WE and EE

ABTS and DPPH methods were utilized to determine the antioxidant capacity of *P. calleryana* fruit WE and EE. As shown in **Table 2**, both WE (2.33 ± 0.15 μg/mL) and EE (2.23 ± 0.15 μg/mL) exhibited significant ABTS radical scavenging effects. Moreover, WE (5.93 ± 0.55 μg/mL) and EE (7.07 ± 0.23 μg/mL) displayed strong DPPH radical scavenging abilities, which was superior or equivalent to that of BHT (7.47 ± 0.47 μg/mL). The DPPH and ABTS free radical scavenging abilities of WE (601.75 ± 55.63 mg AEs/g and 630.35 ± 40.50 mg AEs/g, respectively) were superior or equivalent to those of EE (503.07 ± 17.34 mg AEs/g and 658.75 ± 44.19 mg AEs/g, respectively).

**Table 2:** Antioxidant properties of *P. calleryana* fruit WE and EE.

Samples	ABTS		DPPH	
	IC_50_ (μg/mL)	mg AEs/g sample	IC_50_ (μg/mL)	mg AEs/g sample
WE	2.33 ± 0.15 ^a^	630.35 ± 40.50 ^a^	5.93 ± 0.55 ^a^	601.75 ± 55.63 ^a^
EE	2.23 ± 0.15 ^a^	658.75 ± 44.19 ^a^	7.07 ± 0.23 ^b^	503.07 ± 17.34 ^b^
Ascorbic acid	1.47 ± 0.21 ^b^		3.55 ± 0.07 ^c^	
BHT	1.03 ± 0.25 ^c^		7.47 ± 0.47 ^b^	

Significant differences are indicated by different letters (a-c) in the same column (*p* < 0.05). IC_50_ means the concentration of the sample with a 50% radical scavenging effect. “mg AEs/g sample” indicates milligrams ascorbic acid equivalents per gram of sample.

BHT is widely utilized as a common antioxidant and preservative in the cosmetic and food industries (Yehye et al., 2015). However, toxic effects on the liver, kidneys, and lungs have been reported from prolonged exposure to BHT, raising concerns among consumers (Ghosh et al., 2020). In this study, WE and EE exhibited DPPH radical scavenging abilities that were superior to or comparable to BHT. Therefore, *P. calleryana* fruit WE and EE can be used as antioxidant substitutes for BHT. The determination of TPC and TFC indicated that phenolic and flavonoid compounds were abundant in *P. calleryana* fruit WE and EE. Plant-derived phenolic and flavonoid compounds have been shown to have potent antioxidant activity (Diaz et al., 2012; Mustafa et al., 2010). In our study, a total of 23 phenols and 13 flavonoids were identified through UHPLC-Q-Orbitrap-MS analysis, among which luteolin (IC_50_: 0.59 ± 0.02 μg/mL and 2.10 ± 0.06 μg/mL, respectively) has been shown to be superior to that of BHT (IC_50_: 1.45 ± 0.02 μg/mL and 10.5 ± 0.08 μg/mL, respectively) and ascorbic acid (IC_50_: 2.16 ± 0.03 μg/mL and 3.03 ± 0.12 μg/mL, respectively) in ABTS and DPPH scavenging effects (Tian et al., 2021a). In the ABTS radical clearance test, vanillin exhibited greater activity than ascorbic acid (Tai et al., 2011). Moreover, ursolic acid (Do Nascimento et al., 2014), vanillic acid (Szwajgier et al., 2005; Tai et al., 2012), isorhamnetin (Gong et al., 2020), morin (Hsu et al., 2022), dehydrodiisoeugenol (Li et al., 2020), 6-gingerol (Mi et al., 2016), caffeic acid (Gülçin, 2006), 3,5-dicaffeoylquinic acid (Hong et al., 2015), ellagic acid (Han et al., 2006), and cynaroside (Zou et al., 2018) have been found to have high ABTS and/or DPPH free radical scavenging capacity. Thus, the strong antioxidant activity of *P. calleryana* fruit WE and EE may be explained by their high content of flavonoids and phenolic compounds. Our results showed that *P. calleryana* fruit could be used as a potential antioxidant in functional foods.

### 3.3 Anti-inflammatory effect in LPS-stimulated RAW264.7 cells of *P. calleryana* fruit WE and EE

#### 3.3.1 Cytotoxicity of *P. calleryana* fruit WE and EE

**Figure 2:**
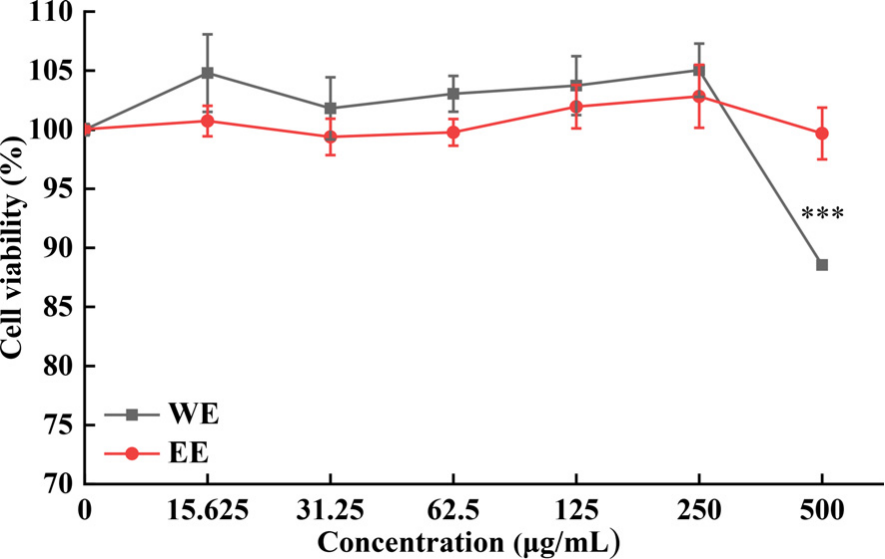
Cytotoxic activity of *P. calleryana* fruit WE and EE on RAW264.7 macrophages. ***p < 0.001 versus the untreated cells.

The MTT assay was used to assess the cytotoxicity of WE and EE against RAW264.7 cells. As shown in **Figure 2**, both WE and EE were non-cytotoxic to RAW264.7 cells in the dose range of 15.625 to 250 mg/mL compared with untreated cells (*p* > 0.05). Therefore, the concentrations of 62.5, 125, and 250 μg/mL were selected for subsequent experiments.

#### 3.3.2 Effects of WE and EE on LPS-induced morphological changes and inflammatory factors release in RAW264.7 cells

**Figure 3:**
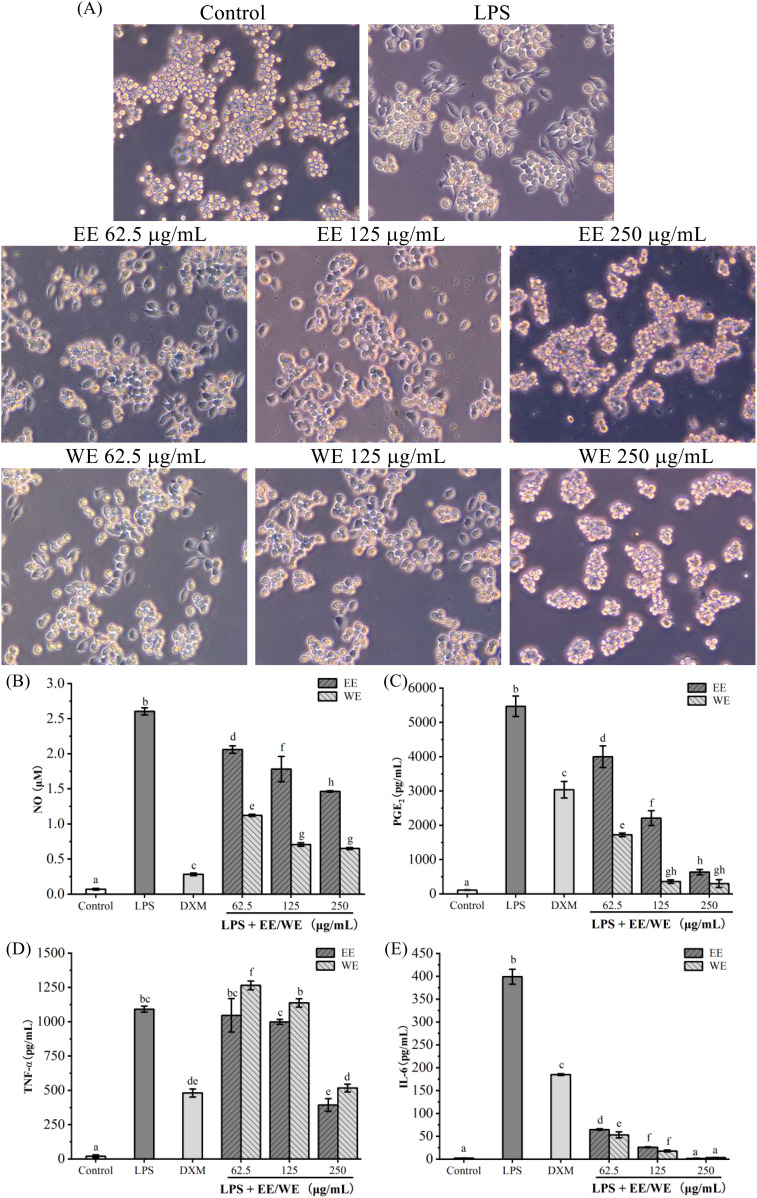
The influence of *P. calleryana* fruit WE and EE on LPS-induced RAW264.7 macrophage morphological alterations and pro-inflammatory mediator and cytokine level. **(A)** *P. calleryana* fruit WE and EE’s effect on alterations in cellular morphology. **(B–E)** The NO release was determined by the NO detection kit, while the levels of PGE_2_, TNF-α, and IL-6 were measured by ELISA kits. The mean ± SD is used to express data. Significant differences are represented by different alphabets (*p* < 0.05).

The pathogenesis of inflammation is closely related to the excessive secretion of pro-inflammatory factors (IL-6 and TNF-α) and mediators (NO and PGE_2_). Inhibiting their production is one of the most important means for treating inflammatory diseases (Wang et al., 2020; Zhao et al., 2021). As shown in **Figure 3A**, the morphology of RAW264.7 cells changed from round to stretched after LPS stimulation compared with control, while the stretched cells gradually decreased with the gradual increase of WE and EE concentrations, especially at high concentrations (250 μg/mL) there were no stretched cells. As shown in **Figures 3B–E**, LPS induction significantly increased the release of NO, PGE_2_, TNF-α, and IL-6. Compared to the LPS group, different concentrations of *P. calleryana* fruit WE and EE (62.5, 125, and 250 μg/mL) markedly suppressed the release of NO, PGE_2_, and IL-6 in RAW264.7 cells induced by LPS, and the high concentration (250 μg/mL) of WE and EE effectively suppressed the level of TNF-α. Moreover, WE (62.5, 125, and 250 μg/mL) and EE (125 and 250 μg/mL) showed better inhibition on PGE_2_ and IL-6 release compared with the positive control DXM (20 μg/mL), and the inhibitory effects of WE on NO, PGE_2_, and IL-6 was superior to or equivalent to that of EE at the same concentrations. These results showed that the *P. calleryana* fruit WE and EE effectively inhibited the level of NO, PGE_2_, TNF-α, and IL-6 in RAW264.7 cells induced by LPS.

Plant-derived flavonoids, terpenoids, and phenolic compounds have been widely demonstrated to be associated with anti-inflammatory effects (Diaz et al., 2012; Faisal et al., 2022; Jaroš et al., 2022; Maleki et al., 2019; Rathee et al., 2009). Phenols, flavonoids, and terpenoids identified in the *P. calleryana* fruit, like ellagic acid, caffeic acid, ursolic acid, and luteolin, reduced the levels of IL-6 and TNF-α in mouse models (Aziz et al., 2018; Chao et al., 2010; Zhao et al., 2023). Besides, 18β-glycyrrhetintic acid significantly reduced TNF-α level in serum and NO level in the kidney of MTX-induced rats (Abd El-Twab et al., 2016). Additionally, in the past *in vitro* studies, vanillin (Zhao et al., 2019), chlorogenic acid (Huang et al., 2023; Hwang et al., 2014), cryptochlorogenic acid (Zhao et al., 2020b), medicarpin (Chern et al., 2021; Mansoori et al., 2020), ethyl caffeate (Chiang et al., 2005), cynaroside (Zou et al., 2018), roburic acid (Islam, 2017), ursonic acid (Son and Lee, 2020), echinocystic acid (Hyam et al., 2013), ursolic acid (Zhao et al., 2023), and 3,5-dicaffeoylquinic acid (Hong et al., 2015) significantly inhibited the release of inflammatory factors, such as TNF-α, IL-6, NO, and PGE_2_. Therefore, the strong anti-inflammatory effects of *P. calleryana* fruit WE and EE may be associated with these flavonoids, terpenoids, and phenolic compounds. Our results suggested that *P. calleryana* fruit could be exploited greatly as an anti-inflammatory agent in functional foods.

### 3.4 Enzyme inhibitory activities of *P. calleryana* fruit WE and EE

Inhibiting the activity of α-glucosidase can suppress oligosaccharide hydrolysis and reduce the absorption of carbohydrates after meals, which can effectively help lower blood glucose (Gao et al., 2022). As shown in **Table 3**, WE (0.60 ± 0.09 μg/mL) and EE (0.48 ± 0.09 μg/mL) exhibited extremely strong α-glucosidase inhibition, which was stronger than that of acarbose (302.57 ± 22.09 μg/mL) (*p* < 0.05). Plant-derived flavonoids and phenolic compounds can serve as natural α-glucosidase inhibitors (Goncçalves and Romano, 2017; Şöhretoğlu and Sari, 2020). Phenolic and flavonoid compounds identified in WE and EE of *P. calleryana* fruit, such as vanillic acid (Singh et al., 2022), vanillin (Zabad et al., 2019), morin (Vanitha et al., 2014), ellagic acid (Chao et al., 2010), isorhamnetin (Alqudah et al., 2023), and luteolin (Zang et al., 2016) have been shown to markedly improved blood glucose levels in a diabetic mouse model. Furthermore, the inhibitory activities of protocatechualdehyde and 6-gingerol on α-glucosidase were superior to those of the positive control acarbose *in vitro* (Mohammed et al., 2017; Tian et al., 2021b). The above findings indicated that *P. calleryana* fruit can be utilized in functional foods as an α-glucosidase inhibitor.

**Table 3:** Enzyme inhibition properties of *P. calleryan* fruit WE and EE.

Samples	Enzyme inhibitory activity (IC_50_, μg/mL)			
	Tyrosinase	α-Glucosidase	Acetylcholinesterase	Butyrylcholinesterase
EE	45.35 ± 0.96 ^a^	0.48 ± 0.09 ^a^	40.47 ± 5.22 ^a^	26.77 ± 0.89 ^a^
WE	210.11 ± 2.59 ^b^	0.60 ± 0.09 ^a^	60.75 ± 5.05 ^b^	34.31 ± 1.97 ^a^
Arbutin	243.07 ± 15.91 ^c^			
Acarbose		302.57 ± 22.09 ^b^		
Galanthamin			0.20 ± 0.01 ^c^	6.38 ± 0.20 ^b^

IC_50_: The concentration of samples that affords a 50% inhibition. Significant differences in the same column are represented by different letters (a-c) (*p* < 0.05).

Tyrosinase is the key enzyme that produces melanin and causes skin pigmentation, and excessive melanin leads to many skin diseases, such as age spots, malignant melanoma, and freckles (Abdul Karim et al., 2014; Brenner and Hearing, 2008). Besides, tyrosinase causes enzymatic browning of beverages, fruits, and vegetables (Loizzo et al., 2012). Therefore, inhibition of tyrosinase activity is useful for achieving skin whitening effects and preventing enzymatic browning of food products. As shown in **Table 3**, the inhibitory effects of WE (210.11 ± 2.59 μg/mL) and EE (45.35 ± 0.96 μg/mL) on tyrosinase were significantly superior to the positive control arbutin (243.07 ± 15.91 μg/mL) (*p* < 0.05). Past studies have shown that phenolic and flavonoid compounds can serve as important sources of tyrosinase inhibitors (Goncçalves and Romano, 2017). Luteolin, a compound identified from the fruits of *P. calleryana*, dose-dependently inhibited tyrosinase activity and melanogenesis in α-melanocyte-stimulating hormone (α-MSH)-stimulated B16 melanoma cells (An et al., 2008; Choi et al., 2008). In addition, benzoic acid (Sima et al., 2011), vanillic acid (Smeriglio et al., 2019), ellagic acid (Pillaiyar et al., 2017), and 2-hydroxy-4-methoxybenzaldehyde (Rafiee and Javaheri, 2015) have been well demonstrated to have good tyrosinase inhibitory effects. Several chemical components identified in *P. calleryana* fruit EE but not detected in WE were proven to have tyrosinase inhibitory effects. For example, ursolic acid exhibited significant anti-tyrosinase effort and inhibited α-MSH-stimulated melanin synthesis in B16F1 cells (Neimkhum et al., 2021; Park et al., 2020). Hesperetin observably inhibited tyrosinase in a competitive manner with IC_50_ = 11.25 ± 1.73 mM (Si et al., 2012). Besides, isorhamnetin (Yu et al., 2019) and morin (Wang et al., 2014) have been demonstrated to serve as tyrosinase inhibitors. These compounds identified only from *P. calleryana* fruit EE may be the reason why EE was significantly superior to WE on tyrosinase inhibition. Hence, the strong tyrosinase inhibition activity of *P. calleryana* fruit WE and EE may be closely related to the presence of these phenolic and flavonoid compounds. The results demonstrated that *P. calleryana* fruit can be developed as a potential tyrosinase inhibitor for skin whitening and prevention of food browning.

Low levels of acetylcholine in the human brain play a crucial role in the pathogenesis of Alzheimer’s disease, and reducing the catabolism of acetylcholine by inhibiting the activities of acetylcholinesterase and butyrylcholinesterase is an effective treatment for Alzheimer’s disease (Adewusi et al., 2010). As shown in **Table 3**, EE (40.47 ± 5.22 μg/mL and 26.77 ± 0.89 μg/mL, respectively) and WE (60.75 ± 5.05 μg/mL and 34.31 ± 1.97 μg/mL, respectively) showed moderate inhibitory effects on acetylcholinesterase and butyrylcholinesterase. Past studies have shown that vanillin and vanillic acid inhibited acetylcholinesterase and butyrylcholinesterase activities (Salau et al., 2020). Besides, 2-hydroxy-4-methoxybenzaldehyde and protocatechualdehyde had potential acetylcholinesterase inhibitory activity (Aderogba et al., 2013; Kundu and Mitra, 2013). These compounds may contribute to the moderate cholinesterase inhibitory activity observed in *P. calleryana* fruit.

## 4 Conclusions

The present study reports the chemical composition, antioxidant, anti-inflammatory, and enzyme inhibitory activities of *P. calleryana* fruit WE and EE for the first time. The chemical composition of *P. calleryana* fruit WE and EE were analyzed by UHPLC-Q-Orbitrap-MS, and the results showed that *P. calleryana* fruit was rich in phenolic, flavonoid, and terpenoid compounds. Both WE and EE exhibited significant free radical scavenging effects on ABTS and DPPH, and their DPPH radical scavenging effects were superior or equivalent to that of BHT. In addition, WE and EE exhibited remarkable anti-inflammatory activity with significant inhibition on the release of pro-inflammatory cytokines (IL-6 and TNF-α) and mediators (NO and PGE_2_) in LPS-stimulated RAW264.7 cells. In enzyme inhibitory activity, *P. calleryana* fruit WE and EE had significant inhibitory effects on tyrosinase and α-glucosidase, whose inhibitory effect was considerably superior to that of the positive control arbutin and acarbose. Our findings showed that *P. calleryana* fruit WE and EE possessed significant antioxidant, anti-inflammatory, α-glucosidase and tyrosinase inhibitory properties. Thus, *P. calleryana* fruit can act as a natural source of antioxidants, anti-inflammatory agents, and tyrosinase and α-glucosidase inhibitors, which has a great potential for application in the field of functional foods. Interestingly, *P. calleryana* fruit exhibited potent inhibition of tyrosinase and α-glucosidase, indicating that it possesses whitening and hypoglycemic efficacy. Further studies should be conducted on its whitening and hypoglycemic effects *in vitro* and *in vivo* to promote its development and utilization in the whitening cosmetics and health food industries.

## Data Availability

The original contributions presented in the study are included in the article/**Supplementary Material**. Further inquiries can be directed to the corresponding author/s.

## References

[B1] Abd El-Twab S. M. Hozayen W. G. Hussein O. E. Mahmoud A. M. (2016). 18β-Glycyrrhetinic acid protects against methotrexate-induced kidney injury by up-regulating the Nrf2/ARE/HO-1 pathway and endogenous antioxidants. Ren. Fail. 38, 1516–1527. doi: 10.1080/0886022X.2016.1216722 27499091

[B2] Abdul Karim A. Azlan A. Ismail A. Hashim P. Abd Gani S. S. Zainudin B. H.. (2014). Phenolic composition, antioxidant, anti-wrinkles and tyrosinase inhibitory activities of cocoa pod extract. BMC Complement. Altern. Med. 14, 381. doi: 10.1186/1472-6882-14-381 25292439 PMC4195981

[B3] Aderogba M. A. Ndhlala A. R. Van Staden J. (2013). Acetylcholinesterase inhibitory activity and mutagenic effects of *Croton penduliflorus* leaf extract constituents. South Afr. J. Bot. 87, 48–51. doi: 10.1016/j.sajb.2013.03.013

[B4] Adewusi E. A. Moodley N. Steenkamp V. (2010). Medicinal plants with cholinesterase inhibitory activity: a review. Afr. J. Biotechnol. 9, 8257–8276. doi: 10.5897/AJB10.1129

[B5] Alqudah A. Qnais E. Y. Wedyan M. A. Altaber S. Bseiso Y. Oqal M.. (2023). Isorhamnetin reduces glucose level, inflammation, and oxidative stress in high-fat diet/streptozotocin diabetic mice model. Molecules 28, 502. doi: 10.3390/molecules28020502 36677559 PMC9866402

[B6] An S. M. Kim H. J. Kim J. E. Boo Y. C. (2008). Flavonoids, taxifolin and luteolin attenuate cellular melanogenesis despite increasing tyrosinase protein levels. Phytother. Res. 22, 1200–1207. doi: 10.1002/ptr.2435 18729255

[B7] Aziz N. Kim M. Y. Cho J. Y. (2018). Anti-inflammatory effects of luteolin: a review of *in vitro*, *in vivo*, and in silico studies. J. Ethnopharmacol. 225, 342–358. doi: 10.1016/j.jep.2018.05.019 29801717

[B8] Brenner M. Hearing V. J. (2008). The protective role of melanin against UV damage in human skin. Photochem. Photobiol. 84, 539–549. doi: 10.1111/j.1751-1097.2007.00226.x 18435612 PMC2671032

[B9] Chao C. Y. Mong M. C. Chan K. C. Yin M. C. (2010). Anti*-*glycative and anti*-*inflammatory effects of caffeic acid and ellagic acid in kidney of diabetic mice. Mol. Nutr. Food Res. 54, 388–395. doi: 10.1002/mnfr.200900087 19885845

[B10] Chern C. M. Lu C. K. Liou K. T. Wang Y. H. Tsai K. C. Chang C. L.. (2021). Medicarpin isolated from Radix Hedysari ameliorates brain injury in a murine model of cerebral ischemia. J. Food Drug Anal. 29, 581–605. doi: 10.38212/2224-6614.3377 35649147 PMC9931010

[B11] Chiang Y. M. Lo C. P. Chen Y. P. Wang S. Y. Yang N. S. Kuo Y. H.. (2005). Ethyl caffeate suppresses NF-κB activation and its downstream inflammatory mediators, iNOS, COX-2, and PGE2 *in vitro* or in mouse skin. Br. J. Pharmacol. 146, 352–363. doi: 10.1038/sj.bjp.0706343 16041399 PMC1576288

[B12] China Medical Information Platform (2024). Pyrus calleryana. Available online at: https://www.dayi.org.cn/cmedical/1116678 (Accessed 6 September 2024).

[B13] Choi M. Y. Song H. S. Hur H. S. Sim S. S. (2008). Whitening activity of luteolin related to the inhibition of cAMP pathway in α-MSH-stimulated B16 melanoma cells. Arch. Pharm. Res. 31, 1166–1171. doi: 10.1007/s12272-001-1284-4 18806960

[B14] Culley T. M. Hardiman N. A. (2007). The beginning of a new invasive plant: a history of the ornamental Callery pear in the United States. BioScience 57, 956–964. doi: 10.1641/B571108

[B15] Diaz P. Jeong S. C. Lee S. Khoo C. Koyyalamudi S. R. (2012). Antioxidant and anti-inflammatory activities of selected medicinal plants and fungi containing phenolic and flavonoid compounds. Chin. Med. 7, 1–9. doi: 10.1186/1749-8546-7-26 23176585 PMC3577437

[B16] Do Nascimento P. G. Lemos T. L. Bizerra A. M. Arriaga Â.M. Ferreira D. A. Santiago G. M.. (2014). Antibacterial and antioxidant activities of ursolic acid and derivatives. Molecules 19, 1317–1327. doi: 10.3390/molecules19011317 24451251 PMC6271190

[B17] Faisal S. Jan H. Abdullah Alam I. Rizwan M. Hussain Z.. (2022). *In vivo* analgesic, anti-inflammatory, and anti-diabetic screening of *Bacopa monnieri*-synthesized copper oxide nanoparticles. ACS Omega 7, 4071–4082. doi: 10.1021/acsomega.1c05410 35155901 PMC8829860

[B18] Gao Y. Bian W. Fang Y. Du P. Liu X. Zhao X.. (2022). [amp]]alpha;-Glucosidase inhibitory activity of fermented okara broth started with the strain *Bacillus amyloliquefaciens* SY07. Molecules 27, 1127. doi: 10.3390/molecules27031127 35164396 PMC8839212

[B19] Ghosh C. Singh V. Grandy J. Pawliszyn J. (2020). Development and validation of a headspace needle-trap method for rapid quantitative estimation of butylated hydroxytoluene from cosmetics by hand-portable GC-MS. RSC Adv. 10, 6671–6677. doi: 10.1039/C9RA08676E

[B20] Goncçalves S. Romano A. (2017). “Inhibitory properties of phenolic compounds against enzymes linked with human diseases,” in Phenolic Compounds-Biological Activity. Eds. Soto-Hernández M. Palma-Tenango M. Garcia-Mateos R. (InTech, London), 99–118.

[B21] Gong G. Guan Y. Y. Zhang Z. L. Rahman K. Wang S. J. Zhou S.. (2020). Isorhamnetin: a review of pharmacological effects. Biomed. Pharmacother. 128, 110301. doi: 10.1016/j.biopha.2020.110301 32502837

[B22] Gülçin I. (2006). Antioxidant activity of caffeic acid (3,4-dihydroxycinnamic acid). Toxicology 217, 213–220. doi: 10.1016/j.tox.2005.09.011 16243424

[B23] Han D. H. Lee M. J. Kim J. H. (2006). Antioxidant and apoptosis-inducing activities of ellagic acid. Anticancer Res. 26, 3601–3606. 17094489

[B24] Hong S. Joo T. Jhoo J. W. (2015). Antioxidant and anti-inflammatory activities of 3, 5-dicaffeoylquinic acid isolated from *Ligularia fischeri* leaves. Food Sci. Biotechnol. 24, 257–263. doi: 10.1007/s10068-015-0034-y

[B25] Hsu J. H. Yang C. S. Chen J. J. (2022). Antioxidant, anti-α-glucosidase, antityrosinase, and anti-inflammatory activities of bioactive components from *Morus alba* . Antioxidants 11, 2222. doi: 10.3390/antiox11112222 36421408 PMC9686747

[B26] Huang J. Xie M. He L. Song X. Cao T. (2023). Chlorogenic acid: a review on its mechanisms of anti-inflammation, disease treatment, and related delivery systems. Front. Pharmacol. 14. doi: 10.3389/fphar.2023.1218015 PMC10534970 37781708

[B27] Hwang S. J. Kim Y. W. Park Y. Lee H. J. Kim K. W. (2014). Anti-inflammatory effects of chlorogenic acid in lipopolysaccharide-stimulated RAW 264.7 cells. Inflamm. Res. 63, 81–90. doi: 10.1007/s00011-013-0674-4 24127072

[B28] Hyam S. R. Jang S. E. Jeong J. J. Joh E. H. Han M. J. Kim D. H. (2013). Echinocystic acid, a metabolite of lancemaside A, inhibits TNBS-induced colitis in mice. Int. Immunopharmacol. 15, 433–441. doi: 10.1016/j.intimp.2012.12.017 23352442

[B29] Islam M. T. (2017). Pharmacological activities of roburic acid: A review of literature. Int. J. Adv. Biochem. Res. 1, 26–28. doi: 10.33545/26174693.2017.v1.i2a.110

[B30] Jaroš P. Vrublevskaya M. Lokočová K. Michailidu J. Kolouchová I. Demnerová K. (2022). *Boswellia serrata* extract as an antibiofilm agent against *Candida* spp. Microorganisms 10, 171. doi: 10.3390/microorganisms10010171 35056620 PMC8778954

[B31] Jiang S. Zheng X. Yu P. Yue X. Ahmed M. Cai D.. (2016). Primitive genepools of Asian pears and their complex hybrid origins inferred from fluorescent sequence-specific amplification polymorphism (SSAP) markers based on LTR retrotransposons. PloS One 11, e0149192. doi: 10.1371/journal.pone.0149192 26871452 PMC4752223

[B32] Kundu A. Mitra A. (2013). Flavoring extracts of *Hemidesmus indicus* roots and *Vanilla planifolia* pods exhibit *in vitro* acetylcholinesterase inhibitory activities. Plant Foods Hum. Nutr. 68, 247–253. doi: 10.1007/s11130-013-0363-z 23715789

[B33] Li C. W. Chu Y. C. Huang C. Y. Fu S. L. Chen J. J. (2020). Evaluation of antioxidant and anti-α-glucosidase activities of various solvent extracts and major bioactive components from the seeds of *Myristica fragrans* . Molecules 25, 5198. doi: 10.3390/molecules25215198 33171671 PMC7664639

[B34] Li H. Cong Y. Lin J. Chang Y. (2015). Enhanced tolerance and accumulation of heavy metal ions by engineered *Escherichia coli* expressing *Pyrus calleryana* phytochelatin synthase. J. Basic Microbiol. 55, 398–405. doi: 10.1002/jobm.201300670 25727053

[B35] Liu X. H. Yao L. Qin Y. R. Liu Z. Q. Yu C. H. (2011). Preliminary on the extraction technics conditions of polysaccharide from *Pyrus calleryana* Decne. Food Sci. Technol. 36, 159–163. doi: 10.13684/j.cnki.spkj.2011.03.023

[B36] Loizzo M. R. Tundis R. Menichini F. (2012). Natural and synthetic tyrosinase inhibitors as antibrowning agents: an update. Compr. Rev. Food Sci. Food Saf. 11, 378–398. doi: 10.1111/j.1541-4337.2012.00191.x

[B37] Maleki S. J. Crespo J. F. Cabanillas B. (2019). Anti-inflammatory effects of flavonoids. Food Chem. 299, 125124. doi: 10.1016/j.foodchem.2019.125124 31288163

[B38] Mansoori M. N. Raghuvanshi A. Shukla P. Awasthi P. Trivedi R. Goel A.. (2020). Medicarpin prevents arthritis in post-menopausal conditions by arresting the expansion of TH17 cells and pro-inflammatory cytokines. Int. Immunopharmacol. 82, 106299. doi: 10.1016/j.intimp.2020.106299 32097846

[B39] Mi H. Guo X. Li J. (2016). Effect of 6-gingerol as natural antioxidant on the lipid oxidation in red drum fillets during refrigerated storage. LWT-Food Sci. Technol. 74, 70–76. doi: 10.1016/j.lwt.2016.07.029

[B40] Mohammed A. Gbonjubola V. A. Koorbanally N. A. Islam M. S. (2017). Inhibition of key enzymes linked to type 2 diabetes by compounds isolated from *Aframomum melegueta* fruit. Pharm. Biol. 55, 1010–1016. doi: 10.1080/13880209.2017.1286358 28176546 PMC6130490

[B41] Mustafa R. A. Hamid A. A. Mohamed S. Abu Bakar F. (2010). Total phenolic compounds, flavonoids, and radical scavenging activity of 21 selected tropical plants. J. Food Sci. 75, C28–C35. doi: 10.1111/j.1750-3841.2009.01401.x 20492146

[B42] Nassar M. I. Mohamed T. K. El-Toumy S. A. Gaara A. H. El-Kashak W. A. Brouard I.. (2011). Phenolic metabolites from *Pyrus calleryana* and evaluation of its free radical scavenging activity. Carbohydr. Res. 346, 64–67. doi: 10.1016/j.carres.2010.11.007 21130983

[B43] Neimkhum W. Anuchapreeda S. Lin W. C. Lue S. C. Lee K. H. Chaiyana W. (2021). Effects of *carissa carandas* linn. Fruit, pulp, leaf, and seed on oxidation, inflammation, tyrosinase, matrix metalloproteinase, elastase, and hyaluronidase inhibition. Antioxidants 10, 1345. doi: 10.3390/antiox10091345 34572978 PMC8470603

[B44] Park H. J. Jo D. S. Choi D. S. Bae J. E. Park N. Y. Kim J. B.. (2020). Ursolic acid inhibits pigmentation by increasing melanosomal autophagy in B16F1 cells. Biochem. Biophys. Res. Commun. 531, 209–214. doi: 10.1016/j.bbrc.2020.07.125 32792197

[B45] Pillaiyar T. Manickam M. Namasivayam V. (2017). Skin whitening agents: medicinal chemistry perspective of tyrosinase inhibitors. J. Enzyme Inhib. Med. Chem. 32, 403–425. doi: 10.1080/14756366.2016.1256882 28097901 PMC6010116

[B46] Rafiee M. Javaheri M. (2015). A theoretical study of benzaldehyde derivatives as tyrosinase inhibitors using Ab initio calculated NQCC parameters. Mol. Biol. Res. Commun. 4, 151–159. doi: 10.22099/mbrc.2015.3118 27844007 PMC5019207

[B47] Rathee P. Chaudhary H. Rathee S. Rathee D. Kumar V. Kanchan K. (2009). Mechanism of action of flavonoids as anti-inflammatory agents: a review. Inflamm. Allergy-Drug Targets 8, 229–235. doi: 10.2174/187152809788681029 19601883

[B48] Salau V. F. Erukainure O. L. Ibeji C. U. Olasehinde T. A. Koorbanally N. A. Islam M. S. (2020). Vanillin and vanillic acid modulate antioxidant defense system via amelioration of metabolic complications linked to Fe 2+-induced brain tissues damage. Metab. Brain Dis. 35, 727–738. doi: 10.1007/s11011-020-00545-y 32065337

[B49] Santamour F. S. Demuth P. (1980). Identification of Callery pear cultivars by peroxidase isozyme patterns. J. Hered. 71, 447–449. doi: 10.1093/oxfordjournals.jhered.a109412

[B50] Sapkota S. Boggess S. L. Trigiano R. N. Klingeman W. E. Hadziabdic D. Coyle D. R.. (2022). Microsatellite loci reveal high genetic diversity, mutation, and migration rates as invasion drivers of Callery pear (*Pyrus calleryana*) in the Southeastern United States. Front. Genet. 13. doi: 10.3389/fgene.2022.861398 PMC9037086 35480304

[B51] Si Y. X. Wang Z. J. Park D. Chung H. Y. Wang S. F. Yan L.. (2012). Effect of hesperetin on tyrosinase: Inhibition kinetics integrated computational simulation study. Int. J. Biol. Macromol. 50, 257–262. doi: 10.1016/j.ijbiomac.2011.11.001 22093614

[B52] Sima V. H. Patris S. Aydogmus Z. Sarakbi A. Sandulescu R. Kauffmann J. M. (2011). Tyrosinase immobilized magnetic nanobeads for the amperometric assay of enzyme inhibitors: application to the skin whitening agents. Talanta 83, 980–987. doi: 10.1016/j.talanta.2010.11.005 21147347

[B53] Singh B. Kumar A. Singh H. Kaur S. Arora S. Singh B. (2022). Protective effect of vanillic acid against diabetes and diabetic nephropathy by attenuating oxidative stress and upregulation of NF-κB, TNF-α and COX-2 proteins in rats. Phytother. Res. 36, 1338–1352. doi: 10.1002/ptr.7392 35088468

[B54] Smeriglio A. D’Angelo V. Denaro M. Trombetta D. Raimondo F. M. Germanò M. P. (2019). Polyphenol characterization, antioxidant and skin whitening properties of *Alnus cordata* stem bark. Chem. Biodivers. 16, e1900314. doi: 10.1002/cbdv.201900314 31397975

[B55] Son J. Lee S. Y. (2020). Therapeutic potential of ursonic acid: comparison with ursolic acid. Biomolecules 10, 1505. doi: 10.3390/biom10111505 33147723 PMC7693102

[B56] Şöhretoğlu D. Sari S. (2020). Flavonoids as alpha-glucosidase inhibitors: mechanistic approaches merged with enzyme kinetics and molecular modelling. Phytochem. Rev. 19, 1081–1092. doi: 10.1007/s11101-019-09610-6

[B57] Szwajgier D. Pielecki J. Targoński Z. (2005). Antioxidant activities of cinnamic and benzoic acid derivaties. Acta Sci. Pol. Technol. Aliment. 4, 129–142.

[B58] Tai A. Sawano T. Ito H. (2012). Antioxidative properties of vanillic acid esters in multiple antioxidant assays. Biosci. Biotechnol. Biochem. 76, 314–318. doi: 10.1271/bbb.110700 22313772

[B59] Tai A. Sawano T. Yazama F. Ito H. (2011). Evaluation of antioxidant activity of vanillin by using multiple antioxidant assays. Biochim. Biophys. Acta 1810, 170–177. doi: 10.1016/j.bbagen.2010.11.004 21095222

[B60] Tian T. Chen G. Y. Zhang H. Yang F. Q. (2021b). Personal glucose meter for α-glucosidase inhibitor screening based on the hydrolysis of maltose. Molecules 26, 4638. doi: 10.3390/molecules26154638 34361791 PMC8348101

[B61] Tian C. Liu X. Chang Y. Wang R. Lv T. Cui C.. (2021a). Investigation of the anti-inflammatory and antioxidant activities of luteolin, kaempferol, apigenin and quercetin. South Afr. J. Bot. 137, 257–264. doi: 10.1016/j.sajb.2020.10.022

[B62] Tian M. Wang Q. Jia X. Tian Y. Hong Y. Zhou Y. (2023). Chemical constituents, antibacterial and anti-inflammatory properties of *Pyrus calleryana* Dcne. essential oil. Ind. Crop Prod. 204, 117353. doi: 10.1016/j.indcrop.2023.117353

[B63] Vanitha P. Uma C. Suganya N. Bhakkiyalakshmi E. Suriyanarayanan S. Gunasekaran P.. (2014). Modulatory effects of morin on hyperglycemia by attenuating the hepatic key enzymes of carbohydrate metabolism and β-cell function in streptozotocin-induced diabetic rats. Environ. Toxicol. Pharmacol. 37, 326–335. doi: 10.1016/j.etap.2013.11.017 24384280

[B64] Wang Q. Liang J. Stephen Brennan C. Ma L. Li Y. Lin X.. (2020). Anti-inflammatory effect of alkaloids extracted from *Dendrobium aphyllum* on macrophage RAW 264.7 cells through NO production and reduced IL-1, IL-6, TNF-α and PGE2 expression. Int. J. Food Sci. Technol. 55, 1255–1264. doi: 10.1111/ijfs.14404

[B65] Wang Y. Zhang G. Yan J. Gong D. (2014). Inhibitory effect of morin on tyrosinase: insights from spectroscopic and molecular docking studies. Food Chem. 163, 226–233. doi: 10.1016/j.foodchem.2014.04.106 24912720

[B66] Wu G. M. Wu F. H. (2012). Processing technology of fermented *Pyrus calleryana* D. wine. China Brew 31, 162–165.

[B67] Yehye W. A. Rahman N. A. Ariffin A. Abd Hamid S. B. Alhadi A. A. Kadir F. A.. (2015). Understanding the chemistry behind the antioxidant activities of butylated hydroxytoluene (BHT): a review. Eur. J. Med. Chem. 101, 295–312. doi: 10.1016/j.ejmech.2015.06.026 26150290

[B68] Yu Q. Fan L. Duan Z. (2019). Five individual polyphenols as tyrosinase inhibitors: inhibitory activity, synergistic effect, action mechanism, and molecular docking. Food Chem. 297, 124910. doi: 10.1016/j.foodchem.2019.05.184 31253292

[B69] Zabad I. E. M. Amin M. N. El-Shishtawy M. M. (2019). Protective effect of vanillin on diabetic nephropathy by decreasing advanced glycation end products in rats. Life Sci. 239, 117088. doi: 10.1016/j.lfs.2019.117088 31759039

[B70] Zang Y. Igarashi K. Li Y. (2016). Anti-diabetic effects of luteolin and luteolin-7-O-glucoside on KK-A^y^ mice. Biosci. Biotechnol. Biochem. 80, 1580–1586. doi: 10.1080/09168451.2015.1116928 27170065

[B71] Zhao X. Chen Q. Lu T. Wei F. Yang Y. Xie D.. (2020a). Chemical composition, antibacterial, anti-inflammatory, and enzyme inhibitory activities of essential oil from *Rhynchanthus beesianus* rhizome. Molecules 26, 167. doi: 10.3390/molecules26010167 33396533 PMC7795889

[B72] Zhao D. Jiang Y. Sun J. Li H. Huang M. Sun X.. (2019). Elucidation of the anti-inflammatory effect of vanillin in LPS-activated THP-1 cells. J. Food Sci. 84, 1920–1928. doi: 10.1111/1750-3841.14693 31264720

[B73] Zhao M. Wu F. Tang Z. Yang X. Liu Y. Wang F.. (2023). Anti-inflammatory and antioxidant activity of ursolic acid: a systematic review and meta-analysis. Front. Pharmacol. 14. doi: 10.3389/fphar.2023.1256946 PMC10568483 37841938

[B74] Zhao H. Wu L. Yan G. Chen Y. Zhou M. Wu Y.. (2021). Inflammation and tumor progression: signaling pathways and targeted intervention. Signal Transduction Targeting Ther. 6, 263. doi: 10.1038/s41392-021-00658-5 PMC8273155 34248142

[B75] Zhao X. L. Yu L. Zhang S. D. Ping K. Ni H. Y. Qin X. Y.. (2020b). Cryptochlorogenic acid attenuates LPS-induced inflammatory response and oxidative stress via upregulation of the Nrf2/HO-1 signaling pathway in RAW 264.7 macrophages. Int. Immunopharmacol. 83, 106436. doi: 10.1016/j.intimp.2020.106436 32234671

[B76] Zou Y. Zhang M. Zhang T. Wu J. Wang J. Liu K.. (2018). Antioxidant and anti-inflammatory activities of cynaroside from *Elsholtiza bodinieri* . Nat. Prod. Commun. 13, 1501–1504. doi: 10.1177/1934578X1801301122

