# Supplementary material for: Phytochemical analysis, antioxidant, anti-inflammatory and enzyme inhibitory activities of bean pear (Pyrus calleryana fruit)
Source: Front Plant Sci. 2025 Feb 7;16:1521990. doi: 10.3389/fpls.2025.1521990 (PMC11842399; doi:10.3389/fpls.2025.1521990)
Supplement: Supplementary file 1 [file DataSheet1.docx]

**Supplementary Materials**

**Abbreviations:**

WE, water extract; EE, ethanol extract; UHPLC-Q-Orbitrap-MS, ultra-high-performance liquid chromatography coupled to quadrupole-Orbitrap high-resolution mass spectrometry; mg GAEs/g sample, milligrams of gallic acid equivalents per gram of sample; mg REs/g sample, milligrams of rutin equivalents per gram of sample; mg AEs/g sample, milligrams of ascorbic acid equivalents per gram of sample; LPS, lipopolysaccharide; MTT, 3-[4,5-Dimethylthiazol-2-yl]-2, 5-diphenyltetrazolium bromide; DXM, dexamethasone; DMSO, dimethyl sulfoxide; TNF-α, tumor necrosis factor-alpha; PGE_2_, prostaglandin E_2_; IL-6, interleukin-6; NO, nitric oxide; p-NPG, p-nitrophenyl-α-D-glucopyranoside; ATCI, acetylthiocholine iodide; AChE, acetylcholinesterase ; BTCl, butyrylthiocholine chloride; BChE, butyrylcholinesterase; BHT, butylated hydroxytoluene; ABTS, 2,2-azino-bis-3-ethylbenzthiazoline-6-sulphonic acid; DPPH, 1,1-Diphenyl-2-picrylhydrazyl; TPC, total phenolic acid content; TFC, total flavonoid content; PBS, phosphate-buffered saline.

**FIGURE S1** UHPLC-Q-Orbitrap-MS chromatograms of *P. calleryana* fruit WE and EE. A: EE in positive ion mode; B: EE in negative ion mode; C: WE in positive ion mode; D: WE in negative ion mode.

**FIGURE S2** Twenty-three phenolic compounds identified in *P. calleryana* fruit WE and EE

**FIGURE S3** Thirteen flavonoids identified in *P. calleryana* fruit WE and EE

**FIGURE S4** Fourteen terpenoid compounds identified in *P. calleryana* fruit WE and EE

**FIGURE S5** Thirteen other types of compounds identified in *P. calleryana* fruit WE and EE

[1] Sucrose (Valgimigli et al., 2012)

[2] Quinic acid (Deshpande et al., 2016)

[3] γ-Aminobutyric acid (Wojnicz et al., 2016)

[4] L-Tryptophan (Zhang et al., 2019)

[5] Scopolin (Cao et al., 2022)

[6] Neochlorogenic acid (Willems et al., 2016; Szymborska et al., 2022)

[7] Citric acid (Wang and Liu et al., 2019)

[8] 3, 5-Dimethoxy-4-hydroxybenzaldehyde

[9] o-Veratraldehyde (Wang et al., 2021; Ding et al., 2019)

[10] Mannitol (Huang et al., 2020)

[11] Gentisic acid (Cao et al., 2022; Ma et al., 2022)

[12] Cryptochlorogenic acid (Alcázar Magaña et al., 2021; Willems et al., 2016)

[13] 2-Isopropylmalic acid (Menicatti et al., 2020)

[14] Aloenin (Wu et al., 2013)

[15] Chlorogenic acid (Salman et al., 2018; Willems et al., 2016)

[16] Vanillic acid (Ostrowski et al., 2014)

[17] 1-Caffeoylquinic acid (Willems et al., 2016; Xue et al., 2016)

[18] Caffeic acid (Santos et al., 2013)

[19] Homoveratrumic acid

[20] Ailanthone (Chuang et al., 2022)

[21] 7-Hydroxycoumarin (Majnooni et al., 2020; Ren et al., 2016)

[22] (+)-Catechin hydrate (Xu et al., 2021)

[23] Vanillin (Constant et al., 2012; Hertzog et al., 2018; Shen et al., 2014)

[24] 2-Hydroxy-4-methoxybenzaldehyde (Ding et al., 2019)

[25] Sarracenin

[26] Procyanidin B1 (Callemien and Collin, 2008; Heger et al., 2022; Weinert et al., 2012)

[27] Ethyl 4-methoxycinnamate

[28] Protocatechualdehyde (Huang et al., 2020; Kong et al., 2020)

[29] Taxifolin 7-rhamnoside (Abad-García et al., 2009; Yang et al., 2016)

[30] Androsin

[31] Medicarpin (Wang and Li et al., 2019)

[32] Hesperetin (Khan et al., 2020)

[33] Morin (Scigelova et al., 2011)

[34] Ellagic acid (Li et al., 2021)

[35] Cynaroside (Chen et al., 2024)

[36] Sinapyl aldehyde (Silva et al., 2023)

[37] Perillene (Ha et al., 2015)

[38] Dehydrodiisoeugenol (Lv et al., 2017)

[39] 3, 5-Dicaffeoylquinic acid (Yang et al., 2022)

[40] Isochlorogenic acid B

[41] Isorhamnetin (Justesen, 2000)

[42] Benzoic acid (Jia et al., 2023)

[43] Diosmetin-7-O-β-D-glucopyranoside

[44] Isochlorogenic acid C (Yang et al., 2022)

[45] Hyperoside (Ji et al., 2022)

[46] Isoquercitrin

[47] Kaempferol-7-O-β-D-glucopyranoside

[48] Germacrone (Ying et al., 2021)

[49] Ethyl caffeate (Santos et al., 2013)

[50] Luteolin (Ding et al., 2021; Fabre et al., 2001)

[51] Astringin (Moss et al., 2013)

[52] Glabrolide (Ran et al., 2024)

[53] 6-Gingerol

[54] Medicagenic acid (Biazzi et al., 2015; Peeters et al., 2020)

[55] Quillaic acid (Ferreira et al., 2018; Niu et al., 2024)

[56] 18 β-Glycyrrhetintic acid (Fomenko et al., 2022; Kowalska and Kalinowska-Lis, 2019; Musharraf et al., 2013)

[57] Ursonic acid (Ran et al., 2024)

[58] Echinocystic acid (Niu et al., 2024)

[59] Ursolic acid (Novotny et al., 2003)

[60] (+)-Usniacin (Jin et al., 2018; Musharraf et al., 2015; Xu et al., 2017)

[61] Acetyl-11-keto-β-boswellic acid (Katragunta et al., 2019)

[62] Lupenone (Heinzen et al., 1996)

[63] Roburic acid (Ayatollahi et al., 2011)

**References**

Abad-García, B., Garmón–Lobato, S., Berrueta, L. A., Gallo, B., and Vicente, F. (2009). A fragmentation study of dihydroquercetin using triple quadrupole mass spectrometry and its application for identification of dihydroflavonols in *Citrus* juices. *Rapid Commun. Mass Spectrom*. 23, 2785–2792. [doi: 10.1002/rcm.4182](https://doi.org/10.1002/rcm.4182)

Alcázar Magaña, A., Kamimura, N., Soumyanath, A., Stevens, J. F., and Maier, C. S. (2021). Caffeoylquinic acids: chemistry, biosynthesis, occurrence, analytical challenges, and bioactivity. *Plant J.* 107, 1299–1319. doi: 10.1111/tpj.15390

Ayatollahi, A. M., Ghanadian, M., Afsharypour, S., Abdella, O. M., Mirzai, M., and Askari, G. (2011). Pentacyclic triterpenes in *Euphorbia microsciadia* with their T-cell proliferation activity. *Iran. J. Pharm. Res*. 10, 287.

Biazzi, E., Carelli, M., Tava, A., Abbruscato, P., Losini, I., Avato, P., et al. (2015). CYP72A67 catalyzes a key oxidative step in *Medicago truncatula* hemolytic saponin biosynthesis. *Mol. Plant*. 8, 1493–1506. doi: 10.1016/j.molp.2015.06.003

Callemien, D., and Collin, S. (2008). Use of RP-HPLC-ESI (–)-MS/MS to differentiate various proanthocyanidin isomers in lager beer extracts. *J. Am. Soc. Brew. Chem*. 66, 109–115. doi: 10.1094/ASBCJ-2008-0215-01

Cao, S., Hu, M., Yang, L., Li, M., Shi, Z., Cheng, W., et al. (2022). Chemical constituent analysis of *Ranunculus sceleratus* L. using ultra-high-performance liquid chromatography coupled with quadrupole-orbitrap high-resolution mass spectrometry. *Molecules* 27, 3299. doi: 10.3390/molecules27103299

Chen, Y., Dong, Y., Song, L., Bai, C., Wang, B., and Sa, C. (2024). The analysis of *Leontopodium* leontopodioides (Willd.) Beauv. chemical composition by GC/MS and UPLC-Q-Orbitrap MS. *Int. J. Anal. Chem*. 2024, 3525212. doi: 10.1155/2024/3525212

Chuang, L., Liu, S., Biedermann, D., and Franke, J. (2022). Identification of early quassinoid biosynthesis in the invasive tree of heaven (*Ailanthus altissima*) confirms evolutionary origin from protolimonoids. *Front. Plant Sci.* 13, 958138. doi: 10.3389/fpls.2022.958138

Constant, S., Robitzer, M., Quignard, F., and Di Renzo, F. (2012). Vanillin oligomerization as a model of side reactions in lignin fragmentation. *Catal. Today* 189, 123–128. doi: 10.1016/j.cattod.2012.03.056

Deshpande, S., Matei, M. F., Jaiswal, R., Bassil, B. S., Kortz, U., and Kuhnert, N. (2016). Synthesis, structure, and tandem massspectrometric characterization of the diastereomers of quinic acid. *J. Agric. Food Chem.* 64, 7298–7306. doi: 10.1021/acs.jafc.6b02472

Ding, F. J., Liu, J. T., Du, R. K., Yu, Q. H., Gong, L. L., Jiang, H. Q., et al. (2019). Qualitative and quantitative analysis for the chemical constituents of *Tetrastigma hemsleyanum* diels et Gilg using ultra-high performance liquid chromatography/hybrid quadrupole-orbitrap mass spectrometry and preliminary screening for anti-influenza virus components. *Evid. Based. Complement. Alternat*. *Med.*  2019, 9414926. doi: 10.1155/2019/9414926

Ding, Y., Chen, S., Wang, H., Li, S., Ma, C., Wang, J., et al. (2021). Identification of secondary metabolites in *Flammulina velutipes* by UPLC*-*Q*-*Exactive*-*Orbitrap MS. *J. Food Qual.* 2021, 4103952. doi: 10.1155/2021/4103952

Fabre, N., Rustan, I., de Hoffmann, E., and Quetin-Leclercq, J. (2001). Determination of flavone, flavonol, and flavanone aglycones by negative ion liquid chromatography electrospray ion trap mass spectrometry. *J. Am. Soc. Mass Spectrom.* 12, 707–715. doi: 10.1016/S1044-0305(01)00226-4

Ferreira, J. P., Miranda, I., and Pereira, H. (2018). Chemical composition of lipophilic extractives from six *Eucalyptus* barks. *Wood Sci. Technol.* 52, 1685–1699. doi: 10.1007/s00226-018-1054-6

Fomenko, V. V., Rudometova, N. B., Yarovaya, O. I., Rogachev, A. D., Fando, A. A., Zaykovskaya, A. V., et al. (2022). Synthesis and in vitro study of antiviral activity of glycyrrhizin nicotinate derivatives against HIV-1 pseudoviruses and SARS-CoV-2 viruses. *Molecules* 27, 295. doi: 10.3390/molecules27010295

Ha, T. J., Lee, M. H., and Lee, J. H. (2015). Comparison of antioxidant activities and volatile components using GC/MS from leaves of Korean purple perilla (*Perilla frutescens*) grown in a greenhouse.  *Food Sci. Biotechnol.* 24, 1979–1986. doi: 10.1007/s10068-015-0261-2

Heger, T., Zatloukal, M., Kubala, M., Strnad, M., and Gruz, J. (2022).  Procyanidin C1 from *Viola odorata* L. inhibits Na^+^,K^+^-ATPase. *Sci. Rep.* 12, 7011. doi: 10.1038/s41598-022-11086-y

Heinzen, H., de Vries, J. X., Moyna, P., Remberg, G., Martinez, R., and Tietze, L. F. (1996). Mass spectrometry of labelled triterpenoids: thermospray and electron impact ionization analysis. *Phytochem. Anal.* 7, 237–244. doi: 10.1002/(SICI)1099-1565(199609)7:5<237::AID-PCA310>3.0.CO;2-M

Hertzog, J., Carré, V., Dufour, A., and Aubriet, F. (2018). Semi-targeted analysis of complex matrices by ESI FT-ICR MS or how an experimental bias may be used as an analytical tool. *J. Am. Soc. Mass Spectrom.* 29, 543–557. doi: 10.1007/s13361-017-1865-y

Huang, G., Liang, J., Chen, X., Lin, J., Wei, J., Huang, D., et al. (2020). Isolation and identification of chemical constituents from Zhideke Granules by Ultra-Performance liquid chromatography coupled with mass spectrometry. *J. Anal. Methods Chem.* 2020, 8889607. doi: 10.1155/2020/8889607

Ji, L., Shi, W., Li, Y., He, J., Xu, G., Qin, M., et al. (2022). Systematic identification, fragmentation pattern, and metabolic pathways of hyperoside in rat plasma, urine, and feces by UPLC-Q-Exactive Orbitrap MS. *J. Anal. Methods Chem.* 2022, 2623018. doi: 10.1155/2022/2623018

Jia, X., Yang, Y., Wang, Q., Tian, Y., Hong, Y., Tian, M., et al. (2023). Phytochemical composition, antioxidant, anti-tyrosinase, anti-cholinesterase, and anti-inflammatory activities of *Rhynchanthus beesianus* rhizome extracts. *Arab. J. Chem.* 16, 104952. doi: 10.1016/j.arabjc.2023.104952

Jin, Y., Ma, Y., Xie, W., Hou, L., Xu, H., Zhang, K., et al. (2018). UHPLC-Q-TOF-MS/MS-oriented characteristic components dataset and multivariate statistical techniques for the holistic quality control of Usnea. *RSC Adv.* 8, 15487–15500. doi: 10.1039/C8RA00081F

Justesen, U. (2000). Collision-induced fragmentation of deprotonated methoxylated flavonoids, obtained by electrospray ionization mass spectrometry. *J. Mass Spectrom.* 36, 169–178. doi: 10.1002/jms.118

Katragunta, K., Siva, B., Kondepudi, N., Vadaparthi, P. R., Rao, N. R., Tiwari, A. K., et al. (2019). Estimation of boswellic acids in herbal formulations containing *Boswellia serrata* extract and comprehensive characterization of secondary metabolites using UPLC-Q-Tof-MS^e^. *J. Pharm. Anal.* 9, 414–422. doi: 10.1016/j.jpha.2019.09.007

Khan, M., Rauf, W., Fazal-e-Habib, M. R., and Iqbal, M. (2020). Screening and identification of bioactive compounds from citrus against non-structural protein 3 protease of hepatitis C virus genotype 3a by fluorescence resonance energy transfer assay and mass spectrometry. *World J. Hepatol.* 12, 976. doi: 10.4254/wjh.v12.i11.976

Kong, J., Liu, L., Gao, Y., Chen, S., Li, L., Shu, Y., et al. (2020). Characteristic chemical profile of Juhe Fang extract with lipid-lowering properties. *J. Tradit. Chin. Med. Sci.* 7, 233–244. doi: 10.1016/j.jtcms.2020.07.003

Kowalska, A., and Kalinowska-Lis, U. (2019). 18β-Glycyrrhetinic acid: its core biological properties and dermatological applications. *Int. J. Cosmet. Sci.* 2019, 1–7. doi: 10.1111/ics.12548

Li, Y., Li, Y., and Chen, J. (2021). Screening and identification of acetylcholinesterase inhibitors from *Terminalia chebula* fruits based on ultrafiltration and ultra-performance liquid chromatography-quadrupole time-of-flight mass spectrometry. *Microchem. J.* 168, 106438. doi: 10.1016/j.microc.2021.106438

Lv, Q. Q., Yang, X. N., Yan, D. M., Liang, W. Q., Liu, H. N., Yang, X. W., et al. (2017). Metabolic profiling of dehydrodiisoeugenol using xenobiotic metabolomics. *J. Pharm. Biomed. Anal.* 145, 725–733. doi: 10.1016/j.jpba.2017.07.045

Ma, Y., Wang, C., Wang, F., Wang, M., Tian, W., Wu, L., et al. (2022). Rapid identification of chemical components of *Folium Photiniae* based on UPLC-Q-TOF-MS. *Chin. Tradit. Herb. Drug.* 53, 6401–6411.

Majnooni, M. B., Fakhri, S., Shokoohinia, Y., Mojarrab, M., Kazemi-Afrakoti, S., and Farzaei, M. H. (2020). Isofraxidin: synthesis, biosynthesis, isolation, pharmacokinetic and pharmacological properties. *Molecules* 25, 2040. doi: 10.3390/molecules25092040

Menicatti, M., Pallecchi, M., Ricciutelli, M., Galarini, R., Moretti, S., Sagratini, G., et al. (2020). Determination of coeluted isomers in wine samples by application of MS/MS deconvolution analysis. *J. Mass Spectrom.*55, e4607. doi: 10.1002/jms.4607

Moss, R., Mao, Q., Taylor, D., and Saucier, C. (2013). Investigation of monomeric and oligomeric wine stilbenoids in red wines by ultra-high-performance liquid chromatography/electrospray ionization quadrupole time-of-flight mass spectrometry. *Rapid Commun. Mass Spectrom.* 27, 1815–1827. doi: 10.1002/rcm.6636

Musharraf, S. G., Kanwal, N., and Arfeen, Q. U. (2013). Stress degradation studies and stability-indicating TLC-densitometric method of glycyrrhetic acid. *Chem. Cent. J.* 7, 1–10.

Musharraf, S. G., Kanwal, N., Thadhani, V. M., and Choudhary, M. I. (2015). Rapid identification of lichen compounds based on the structure–fragmentation relationship using ESI-MS/MS analysis. *Anal.Methods* 7, 6066–6076. doi: 10.1039/C5AY01091H

Niu, J., Jia, X., Yang, N., Ran, Y., Wu, X., Ding, F., et al. (2024). Phytochemical analysis and anticancer effect of *Camellia oleifera* bud ethanol extract in non-small cell lung cancer A549 cells. *Front. Pharmacol.* 15, 1359632. doi: 10.3389/fphar.2024.1359632

Novotny, L., Abdel-Hamid, M. E., Hamza, H., Masterova, I., and Grancai, D. (2003). Development of LC-MS method for determination of ursolic acid: application to the analysis of ursolic acid in *Staphylea holocarpa* Hemsl. *J. Pharm. Biomed. Anal.* 31, 961–968. doi: 10.1016/S0731-7085(02)00706-9

Ostrowski, W., Wojakowska, A., Grajzer, M., and Stobiecki, M. (2014). Mass spectrometric behavior of phenolic acids standards and their analysis in the plant samples with LC/ESI/MS system. *J. Chromatogr. B. Analyt Technol. Biomed. Life. Sci*. 967, 21–27. doi: 10.1016/j.jchromb.2014.07.005

Peeters, L., Vervliet, P., Foubert, K., Hermans, N., Pieters, L., and Covaci, A. (2020). A comparative study on the in vitro biotransformation of medicagenic acid using human liver microsomes and S9 fractions. *Chem. Biol. Interact.* 328, 109192. doi: 10.1016/j.cbi.2020.109192

Ran, Y., Yang, L., Jia, X., Zhao, H., Hu, Q., Yang, B., et al. (2024). Phytochemical composition and anticancer effect of *Akebia trifoliata* seed in non-small cell lung cancer A549 cells. *Arab. J. Chem.* 17, 106020. doi: 10.1016/j.arabjc.2024.106020

Ren, Z., Nie, B., Liu, T., Yuan, F., Feng, F., Zhang, Y., et al. (2016). Simultaneous determination of coumarin and its derivatives in tobacco products by liquid chromatography-tandem mass spectrometry. *Molecules* 21, 1511. doi: 10.3390/molecules21111511

Salman, H. A., Ramasamy, S., and Mahmood, B. (2018). Detection of caffeic and chlorogenic acids from methanolic extract of *Annona squamosa* bark by LC-ESI-MS/MS. *J. Intercult. Ethnopharmacol.* 7, 76–81. doi: 10.5455/jice.20171011073247

Santos, J. L., Bispo, V. S., Filho, A. B., Pinto, I. F., Dantas, L. S., Vasconcelos, D. F., et al. (2013). Evaluation of chemical constituents and antioxidant activity of coconut water (*Cocus nucifera* L.) and caffeic acid in cell culture. *An. Acad. Bras. Ciênc.* 85, 1235–1247. doi: 10.1590/0001-37652013105312

Scigelova, M., Hornshaw, M., Giannakopulos, A., and Makarov, A. (2011). Fourier transform mass spectrometry. *Mol. Cell. Proteomics*. 10, 009431.

Shen, Y., Han, C., Liu, B., Lin, Z., Zhou, X., Wang, C., et al. (2014). Determination of vanillin, ethyl vanillin, and coumarin in infant formula by liquid chromatography-quadrupole linear ion trap mass spectrometry. *J. Dairy Sci.* 97, 679–686. doi: 10.3168/jds.2013-7308

Silva, D., Sousa, A. C., Robalo, M. P., and Martins, L. O. (2023). A wide array of lignin-related phenolics are oxidized by an evolved bacterial dye-decolourising peroxidase. *N. Biotechnol.* 77, 176–184. doi: 10.1016/j.nbt.2022.12.003

Szymborska, K., Frański, R., and Beszterda-Buszczak, M. (2022). Extraction with acidified methanol—an easy and effective method of methyl chlorogenate formation, as studied by ESI-MS. *Molecules* 27, 7543. doi: 10.3390/molecules27217543

Valgimigli, L., Gabbanini, S., and Matera, R. (2012). “Analysis of maltose and lactose by UHPLC-ESI-MS/MS” in *Dietary Sugars: Chemistry, Analysis, Function and Effects*. Ed R. P. Victor (Cambridge: Royal Society of Chemistry), 443–463.

Wang, H. Y., Li, T., Ji, R., Xu, F., Liu, G. X., Li, Y. L., et al. (2019). Metabolites of medicarpin and their distributions in rats. *Molecules* 24, 1966. doi: 10.3390/molecules24101966

Wang, Z. X., Liu, J. Y., Zhong, X. j., Li, J. J., Wang, X., Ji, L. L.,et al. (2019). Rapid characterization of chemical components in edible mushroom *Sparassis crispa* by UPLC-Orbitrap MS analysis and potential inhibitory effects on Allergic Rhinitis. *Molecules* 24, 3014. doi: 10.3390/molecules24163014

Wang, K., Tian, J., Li, Y., Liu, M., Chao, Y., Cai, Y., et al. (2021). Identification of components in Citri Sarcodactylis Fructus from different origins via UPLC-Q-Exactive Orbitrap/MS. *ACS Omega* 6, 17045–17057. doi: 10.1021/acsomega.1c02124

Weinert, C., Wiese, S., Rawel, H., Esatbeyoglu, T., Winterhalter, P., Homann, T., et al. (2012). Methylation of catechins and procyanidins by rat and human Catechol-O-Methyltransferase: metabolite profiling and molecular modeling studies. *Drug Metab. Dispos.* 40, 353–359. doi: 10.1124/dmd.111.041871

Willems, J. L., Khamis, M. M., Saeid, W. M., Purves, R. W., Katselis, G., Low, N. H., et al. (2016). Analysis of a series of chlorogenic acid isomers using differential ion mobility and tandem mass spectrometry. *Anal. Chim. Acta.* 933, 164–174. doi: 10.1016/j.aca.2016.05.041

Wojnicz, A., Ortiz, J. A., Casas, A. I., Freitas, A. E., López, M. G., and Ruiz-Nuño, A. (2016). Data supporting the rat brain sample preparation and validation assays for simultaneous determination of 8 neurotransmitters and their metabolites using liquid chromatography-tandem mass spectrometry. *Data Brief* 7, 714–720. doi: 10.1016/j.dib.2016.03.025

Wu, X., Ding, W., Zhong, J., Wan, J., and Xie, Z. (2013). Simultaneous qualitative and quantitative determination of phenolic compounds in *Aloe barbadensis* Mill by liquid chromatography-mass spectrometry-ion trap-time-of-flight and high performance liquid chromatography-diode array detector. *J. Pharm. Biomed. Anal.* 80, 94–106. doi: 10.1016/j.jpba.2013.02.034

Xu, M., Heidmarsson, S., Thorsteinsdottir, M., Eiriksson, F. F., Omarsdottir, S., and Olafsdottir, E. S. (2017). DNA barcoding and LC-MS metabolite profiling of the lichen-forming genus *Melanelia*: Specimen identification and discrimination focusing on Icelandic taxa. *PLoS One* 12, e0178012. doi: 10.1371/journal.pone.0178012

Xu, Y., Liang, P. L., Chen, X. L., Gong, M. J., Zhang, L., Qiu, X. H., et al. (2021). The impact of *Citrus*-tea cofermentation process on chemical composition and contents of Pu-Erh Tea: an integrated metabolomics study. *Front. Nutr.* 8, 737539. doi: 10.3389/fnut.2021.737539

Xue, M., Shi, H., Zhang, J., Liu, Q. Q., Guan, J., Zhang, J. Y., et al. (2016). Stability and degradation of caffeoylquinic acids under different storage conditions studied by high-performance liquid chromatography with photo diode array detection and high-performance liquid chromatography with electrospray ionization collision-induced dissociation tandem mass spectrometry. *Molecules* 21, 948. doi: 10.3390/molecules21070948

Yang, P., Xu, F., Li, H. F., Wang, Y., Li, F. C., Shang, M. Y., et al. (2016). Detection of 191 taxifolin metabolites and their distribution in rats using HPLC-ESI-IT-TOF-MS^n^. *Molecules* 21, 1209. doi: 10.3390/molecules21091209

Yang, J., Yao, L., Gong, K., Li, K., Sun, L., and Cai, W. (2022). Identification and quantification of chlorogenic acids from the root bark of *Acanthopanax gracilistylus* by UHPLC-Q-Exactive orbitrap mass spectrometry. *ACS Omega* 7, 25675–25685. doi: 10.1021/acsomega.2c02899

Ying, Y., Yu, M., Xiao, J., and Shen, Q., (2021). Metabolites and metabolic pathway analysis of germacrone in rats by UHPLC-Q-Orbitrap HRMS. *Chin.* *J. Mod. Appl. Pharm.* 38, 430–438.

Zhang, P., Chan, W., Ang, I. L., Wei, R., Lam, M. M., Lei, K. M., et al. (2019). Revisiting fragmentation reactions of protonated α-amino acids by high-resolution electrospray ionization tandem mass spectrometry with collision-induced dissociation. *Sci. Rep.* 9, 6453. doi: 10.1038/s41598-019-42777-8
